# Molecular genetic characterization and meat-use functional gene identification in Jianshui yellow–brown ducks through combined resequencing and transcriptome analysis

**DOI:** 10.3389/fvets.2023.1269904

**Published:** 2023-12-12

**Authors:** Xinpeng Li, Aiguo Xin, Li Ma, Xiao Gou, Suyun Fang, Xinxing Dong, Bin Ni, Lin Tang, Li Zhu, Dawei Yan, Xiaoyan Kong

**Affiliations:** ^1^Faculty of Animal Science and Technology, Yunnan Agricultural University, Kunming, China; ^2^Poultry Husbandry and Disease Research Institute, Yunnan Academy of Animal Husbandry and Veterinary Sciences, Kunming, China; ^3^Animal Husbandry and Veterinary College, Yunnan Vocational and Technical College of Agriculture, Kunming, China; ^4^School of Life Science and Engineering, Foshan University, Foshan, China

**Keywords:** Jianshui yellow–brown duck, meat-use functional gene, molecular genetics, resequencing, transcriptome

## Abstract

The Jianshui yellow–brown duck is a unique country-specific waterfowl species in Yunnan Province, well known for its tender meat. However, there is a lack of comprehensive systematic research on the molecular genetic characteristics, especially germplasm resources and economic traits, of the Jianshui yellow–brown ducks. This study investigated the molecular genetic characteristics of Jianshui yellow–brown ducks, compared their selection signals with those of ancestral mallard and meat-type Pekin ducks, and identified genes specific to their meat-use performance. Furthermore, this study also evaluated the breeding potential for its meat performance. In this study, phylogenetic trees, PCA and Admixture analysis were used to investigate the population genetic structure among local duck breeds in China; population genetic differentiation index (Fst), nucleotide diversity and Tajima’s D were used to detect selected loci and genes in the population of Jianshui yellow–brown ducks; and transcriptome technology was used to screen for differentially expressed genes in the liver, sebum and breast muscle tissues, and finally, the results of the genome selection signals and transcriptome data were integrated to excavate functional genes affecting the meat performance of the Jianshui yellow–brown ducks. The results of the genetic structure of the population showed that Jianshui yellow–brown ducks were clustered into a separate group. Selection signal analysis indicated significant selection pressure on certain genes related to meat characteristics (*ELOVL2*, *ELOVL3*, *GDF10*, *VSTM2A*, *PHOSPHO1*, and *IGF2BP1*) in both Jianshui yellow–brown ducks and mallards. Transcriptomic data analysis suggested that *ELOVL3*, *PHOSPHO1*, and *GDF10* are vital candidate genes influencing meat production and quality in Jianshui yellow–brown ducks. A comparison of selection signals between Jianshui yellow–brown ducks and Pekin ducks revealed only 21 selected genes in the Jianshui yellow–brown duck population, and no significant genes were related to meat traits. Moreover, whole-genome resequencing data suggested that the Jianshui yellow–brown duck represents a unique category with distinct genetic mechanisms. Through selection signaling and transcriptomic approaches, we successfully screened and identified important candidate genes affecting meat traits in Jianshui yellow–brown ducks. Furthermore, the Jianshui yellow–brown duck has good potential for improved meat performance, highlighting the need for further improvement.

## 1 Introduction

China, has an rich extensive history of duck domestication and holds a rich abundance of waterfowl resources. China is home to nearly half of the world’s existing duck varieties (1). However, despite this vast array of varieties, the full potential of the abundant genetic resources remains untapped because of the delayed initiation of modernized breeding practices for domestic ducks in the country. One notable example is the development of Cherry Valley Peking ducks by the British Cherry Valley Company through systematic and scientific breeding of Chinese Peking ducks. In contrast to native Peking ducks, Cherry Valley Peking ducks stand out due to their large size and rapid growth rate. Upon entering the Chinese market, Cherry Valley Peking ducks quickly gained favor among duck farmers and swiftly dominated the Chinese duck meat market. This, in turn, resulted in a significant reduction in the population of native duck breeds and placed substantial pressure on their living spaces (2, 3). Therefore, the rational development and utilization of resources, guided by breed characteristics, are of paramount importance in breed selection and the sustainable development of related industries.

Sequencing technology has been widely used in studying species origin, evaluating germplasm resources, and understanding population genetic diversity. Feng et al. calculated and compared genetic diversity in five duck varieties using genome-wide single-nucleotide polymorphism (SNP) loci. They discovered that domestic duck varieties exhibited lower genetic diversity than wild duck varieties (4). In evolutionary tree analysis, individuals from domestic duck varieties were clustered into one category, while those from wild duck varieties were clustered into another category, suggesting a common wild ancestor for domestic ducks (5). Hence, assessment of the genetic diversity of duck varieties and accurate identification and evaluation of germplasm resources form an important basis for identifying excellent candidate genes and promoting the development, utilization, and innovation of germplasm resources. With the widespread application of high-throughput sequencing technology, the integration of multi-omics data enables a deeper understanding of the genetic basis and molecular mechanisms underlying complex traits (6–8). The lack of systematic breeding conservation measures and scientific selective breeding programs for Jianshui yellow–brown ducks has resulted in issues such as varietal complexity and degradation, thereby affecting its genetic resources adversely.

Jianshui yellow–brown ducks are native to Jianshui County, Yunnan Province, and have excellent traits such as tender meat, docile temperament and strong adaptability after selective breeding. However, as a local breed for meat and eggs, Jianshui yellow–brown ducks, which are less selectively bred, have the potential to be bred into specialized meat breeds (e.g., Pekin ducks for meat breeds) in the future. However, the molecular mechanisms influencing the meat production and quality of Jianshui yellow–brown ducks have not been investigated in depth. This study investigated the population genetic structure and genetic diversity of the Jianshui yellow–brown duck population using resequencing technology. Subsequently, based on the results of the population genetic structure analysis, this study performed a comparison between this duck population and the mallard population to identify candidate genes associated with meat production and fat deposition in Jianshui yellow–brown ducks through combined analysis of selection signals and transcriptomes. Furthermore, a selection signal analysis was performed on Jianshui yellow–brown ducks and meat-type Pekin ducks to explore genomic-level genetic differences between the two varieties resulting from different domestication and breeding processes. Finally, the meat-trait breeding status in Jianshui yellow–brown ducks was evaluated.

## 2 Materials and methods

### 2.1 Sample collection

We randomly selected 18 healthy Jianshui yellow–brown ducks (YB), including 7 males and 11 females, at the Jianshui yellow and brown duck breeding farm in Jianshui County, Yunnan Province. During the collection process, 5 mL of blood samples were taken from the wing veins of these ducks using vacuum blood collection tubes with EDTA anticoagulant. Ice packs were cryopreserved and transported back to the laboratory and stored in a −20°C refrigerator for subsequent extraction of whole gene DNA. In addition, 6 Jianshui yellow–brown ducks (3 males and 3 females) were randomly selected for slaughter, and 6 liver, 6 sebum, and 3 breast muscle tissue samples were collected, immediately place the tissue into a pre-prepared cryopreservation tube containing RNA preservation solution (the RNA preservation solution is RNA solid Stable Preservation Solution, Wuhan Servicebio Company), place it in liquid nitrogen, take it back to the laboratory and store it in a −80°C ultra-low temperature refrigerator for subsequent extraction of total tissue RNA. For detailed information on the collected samples of Jianshui yellow–brown ducks, please refer to Supplementary Table S1. All experimental procedures adhered to the regulations of the Administration of Laboratory Animal Affairs and were approved by the Ethics Committee of Yunnan Agricultural University (No. 202103035).

### 2.2 Whole-genome resequencing and bioinformatics analysis

#### 2.2.1 Population variation detection

The quality and concentration of the extracted DNA were assessed using 1% agarose gel electrophoresis and ultraviolet spectrophotometry. After passing the quality control, the samples will be sent to BerryGenomics Corporation (Beijing, China) for whole genome resequencing. The sequencing will be performed on Illumina novaseq 6,000 sequencing platform, using paired-end sequencing, and the sequencing depth is 15×. Additionally, resequencing data for a few duck varieties were downloaded from the NCBI database (Bioproject ID: PRJNA450892, PRJNA599025, PRJNA645648, and PRJNA419832), including wild varieties (mallard, ML, *n* = 10; spot-billed duck, SB, *n* = 10), meat-type varieties (Pekin duck, PK, *n* = 16; Cherry Valley duck, CV, *n* = 10), and meat-egg-type varieties (Fenghua duck, FH, *n* = 10; Shanma duck, SM, *n* = 10; Shaoxing duck, SX, *n* = 10; Gaoyou duck, GY, *n* = 8; Jinding duck, JD, *n* = 8). Information on some of the Chinese local duck breeds included in this study is shown in Supplementary Table S2. The resequencing data of 18 Jianshui yellow–brown ducks (from a total of 10 populations and 110 individuals) obtained in this study were subjected to population structure and genetic diversity analyses. The raw data underwent quality control and filtering using FASTP (9) software with default parameters. After quality control, the clean data were compared with the Pekin duck reference genome (10) by aligning both sequences using bwa (11) software (version CAU_duck1.0: https://ftp.ensembl.org/pub/release-110/fasta/anas_platyrhynchos_platyrhynchos/dna/Anas_platyrhynchos_platyrhynchos.CAU_duck1.0.dna.toplevel.fa.gz). The sam files were converted to bam files, and sorted using the “-sort” command in SAMTOOLS (12) software. The commands “flagstat” and “coverage” were used to count the alignment rate, coverage rate, and coverage depth in each sample. PICARD software was used to label repetitive sequences resulting from amplification through polymerase chain reaction during sequencing and remove them. Alignment in separate samples and separate chromosomes was conducted using the “HaplotypeCaller,” “CombineGVCFs,” “GenotypeGVCFs,” “MergeVcfs,” and “SelectVariants” commands in the GATK (13) software to obtain the individual variation detection results SNP loci were extracted from the individual results, and population-level SNP variation results were obtained. The population variation detection results were initially filtered using the “VariantFiltration” command with the following specific criteria: QD < 2.0 || MQ < 40.0 || FS > 60.0 || SOR > 3.0 || MQRankSum < −12.5 || ReadPosRankSum < −8.0. All SNPs were further filtered to improve the confidence of the results. Those SNPs that did not meet the requirements of deletion rate and minimum allele frequency in the population were further filtered using VCFTOOLS (14) software according to the criteria of “--geno 0.1 ---maf 0.01.” Finally, the high-confidence population SNP variation detection results were saved in the vcf format and used for subsequent downstream analysis.

#### 2.2.2 Population structure, genetic differentiation, and genetic diversity analyses

Based on the VCF files obtained in the previous step, the population was subjected to PCA analysis using PLINK software, followed by visualization of the PCA results using R. The vcf file of the population SNP variation results was converted to the phylip format using the run_pipeline.pl. program in TASSEL (15) software. A neighbor-joining (NJ) evolutionary tree was constructed using PHYLIP (16) software, and the phylogenetic tree was beautified using the iTOL online website (17). The genetic differentiation index Fst between two populations was calculated using VCFTOOLS software with a sliding window approach, utilizing a window size of 100 kb and a sliding step size of 10 kb. Cross-validation error rates were calculated using ADMIXTURE (18) software for different *K* values, and the optimal *K* value was determined. Stacked plots were created using the pophelper software package in R. Nucleotide diversity values for each of the 10 populations were calculated using the vcftools software “--window-pi 10 M” command, and boxplots were generated using ggplot2 for visualization and comparison [In the study of population genetic differentiation and comparison of population nucleotide diversity, we used the parameters of window size and sliding step size with reference to Wang et al. (19)].

#### 2.2.3 Population selection analysis

Nucleotide diversity ratio θπ and genetic differentiation index Fst were calculated using Vcftools software with a sliding window size of “40 kb” and a sliding step size of “10 kb.” [In the study of selection signal analysis of populations based on population genetic differentiation and population nucleotide diversity, we used the parameters of window size and sliding step size with reference to Zhou et al. (20)]. The top 1% regions of the joint region of θπ and Fst were identified as candidate regions for selection. Duplicate regions were merged using bedtools software, and the genes within the filtered regions were annotated using the species gene annotation file.

### 2.3 Transcriptome sequencing and bioinformatics analysis

The Tiangen Total RNA Extraction Kit was used to extract total RNA from the liver, sebum and breast muscle tissue according to the manufacturer’s instructions. The quality-controlled samples were sent to Berry Genomics (Beijing, China) for sequencing, which will be performed on the Illumina novaseq 6,000 sequencing platform using paired-end sequencing (Paired-end sequencing, measuring 150 bp at each end, and the length of each read is 300 bp) with 10 G per sample (i.e., 1 billion base pairs per sample by transcriptome sequencing to ensure sufficient data for accurate gene expression analysis). The liver, sebum and breast muscle transcriptomic data of mallard and Pekin ducks used in this study were downloaded from public databases (NCBI and BIG Data Center, Bioproject ID: PRJNA645648, BIG accession codes PRJCA001307), transcriptome sequencing data downloaded from public databases also use paired-end sequencing, with 150 bp at each end and a read length of 300 bp. The same tissues from Jianshui yellow–brown ducks, mallards, and Pekin ducks were compared to identify differentially expressed genes (DEGs). The raw data underwent filtering and quality control using FASTP software with default parameters. The resulting clean data were aligned to the Pekin duck reference genes using HISAT2 (21) software (version: CAU_duck1.0). The parameters for mapping sequenced reads back to the reference genome using HISAT2 are as follows: --rna-strandness --new-summary -x genome −1 read1.fq.gz −2 reads2.fq.gz -S sample.sam. We set the parameter “--rna-strandness” mainly because we adopted the strand-specific library construction method when we sequenced the transcriptome of Jianshui yellow–brown duck tissues. In this way, we were able to obtain the orientation information of the RNA fragments to estimate the gene expression level more accurately. At the same time, we used the “-x” parameter to specify the reference gene of the selected species. The aim of this study was to look for differences between species by performing transcriptome analyses of the same tissue from different species. In order to control a single variable as much as possible and reduce the influence of sex factors on differentially expressed genes, we first removed the chromosomal information related to the sex of the species on the reference genome, and only aligned the reads back to the autosomes on the reference genome of the species. Expression quantification was performed on the alignment results using the “featurecounts” command of SUBREAD (22) software to obtain the raw expression matrix. Using the TPM (Transcripts Per Million) approach to normalize the raw expression matrix of transcriptomic data. TPM correction of raw counts prior to correlation analysis of samples is required in order to eliminate variability due to differences in gene length and sequencing depth. This ensures that comparisons of gene expression between samples are accurate and fair. Such standardization is essential for subsequent sample correlation analysis. Differential expression analysis was conducted on the raw expression matrix using DESEQ2 (23). We will use the raw expression matrix generated by featurecounts as the input file for DESEQ2. In this process, we set DESEQ2’s comparison grouping information, i.e., grouping according to varieties. After that DESEQ2 will calculate key information such as log_2_|Foldchange|, value of *p* and padj for each gene. Significant DEGs were identified based on the criteria of |log2 Fold Change| > 1.5 and corrected *p*-values (P.adj) less than 0.001. The correction for false-positive detection due to multiple comparison tests was performed using the Benjamini–Hochberg (BH) method. Screening for differentially expressed genes (DEGs) by adjusted *p*-values reduces the rate of false positives and enhances the reliability of the study results. The number of DEGs in each population was then counted, and volcano plots were generated using R.

Joint analysis of selection signal and transcriptome

To further narrow down the candidate gene list and increase the robustness of the results, a joint analysis of selection signals and transcriptome data was performed. First, we screened the candidate genes related to the target trait (i.e., meat performance) from the selection signal results, and intersected the candidate genes from the selection signal with the differentially expressed genes obtained from the transcriptome analysis, and the intersected portion of the results was the result of the joint analysis.

## 3 Results

### 3.1 Genome sequencing results and variation detection

Eighteen Jianshui yellow–brown ducks underwent resequencing, and the resequencing data of 92 ducks from nine different varieties were downloaded from the NCBI database, totaling 110 individuals from 10 populations. The quality of the sequencing data was assessed, and the 18 Jianshui yellow–brown ducks exhibited a base population quality with Q20 and Q30 values above 90%, normal CG distribution, an average sample alignment rate of 98.22%, an average sequencing depth of 18.94×, and an average sample coverage rate of 92.37% (Supplementary Table S3). The Q20 and Q30 values for the 92 resequencing data from the NCBI database also exceeded 90%, with an average alignment rate of 97.69%, an average sequencing depth of 8.14×, and an average coverage rate of 90.66% (Supplementary Table S4). Thus, the sequencing data demonstrated reliable quality and met the requirements for resequencing analysis. After the detection and filtering of population SNPs, a total of 19,795,912 high-quality SNPs were obtained from 110 individuals. The subsequent analysis focused on population structure, population genetic diversity, and population selection based on these population SNPs.

### 3.2 Population genetic structure and genetic differentiation

**Figure 1:**
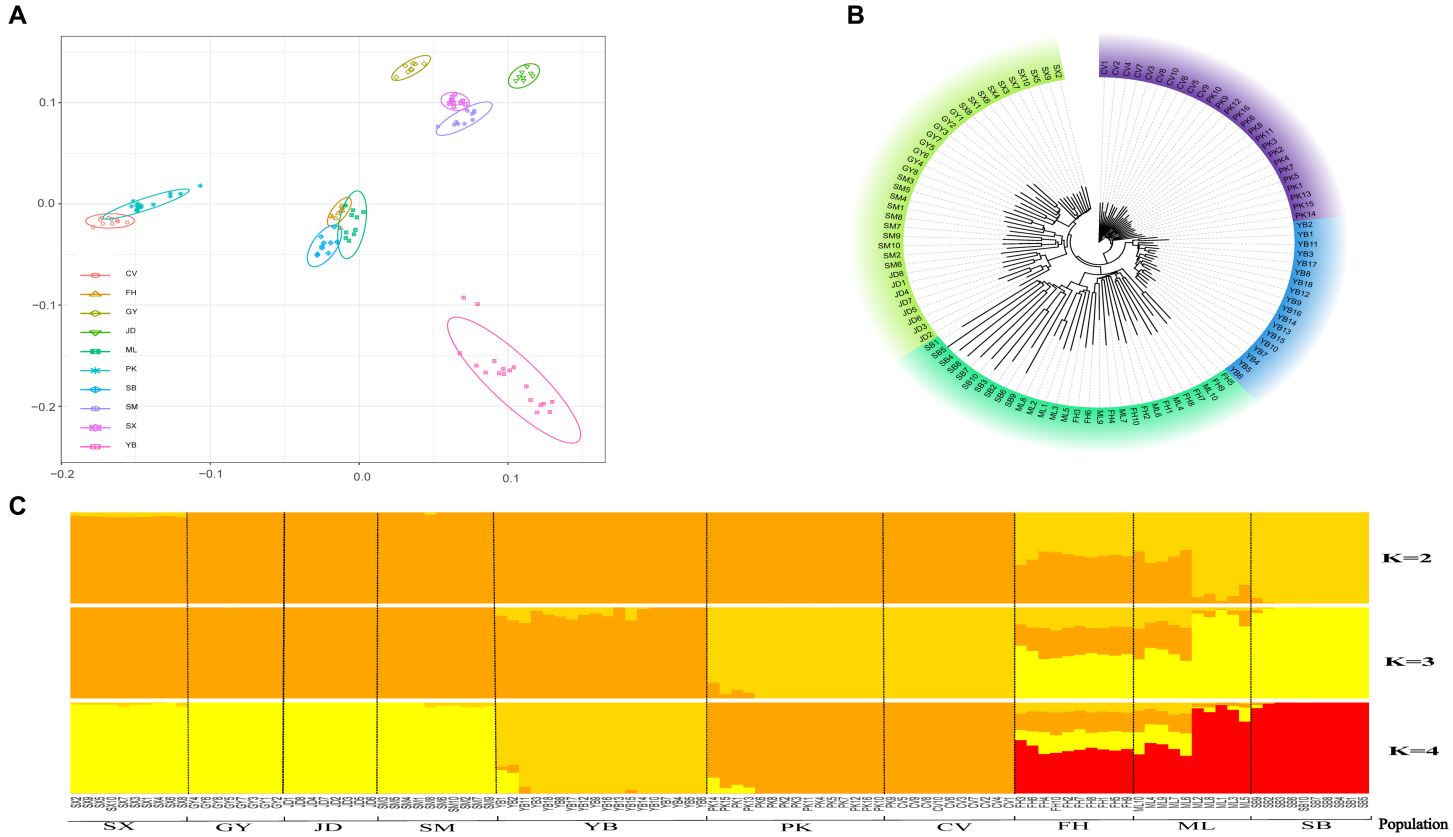
Population stratification of Jianshui yellow–brown duck. **(A)** Principal Component Analysis [Cherry Valley Pekin duck (CV), Fenghua duck (FH), Gaoyou duck (GY), Jinding duck (JD), Mallard (ML), Pekin duck (PK), Spot-billed duck (SB), Shanma duck (SM), Shaoxing duck (SX), Jianshui Yellow–brown duck (YB)]. **(B)** Neighbor-joining phylogenetic of eleven populations. **(C)** Population structure analysis.

To examine the presence of outlier samples and inter-population genetic structure and relationships, PCA, phylogenetic tree construction, and population structure analysis were performed using whole-genome SNPs from 110 individuals representing 10 populations (Pekin, Cherry Valley Pekin, mallard, spot-billed, Fenghua, Shanma, Shaoxing, Jinding, Gaoyou, and Jianshui yellow–brown ducks). PCA results demonstrated that individuals within the same population tended to cluster together (Figure 1A). The 10 populations could be categorized into four groups: Gaoyou duck, Jinding duck, Shanma duck, and Shaoxing duck formed one group; mallard, spot-billed duck, and Fenghua duck formed another group; Pekin duck and Cherry Valley Pekin duck clustered together; and Jianshui yellow–brown duck formed a distinct individual population. The results from the NJ evolutionary tree (Figure 1B) were consistent with the PCA results, with the Jianshui yellow–brown duck population forming a separate cluster. Structure analysis indicated that the cross-validation error rate was minimized when *K* = 4 (Supplementary Figure S1). Figure 1C depicts the structure results when *K* = 2–4. When *K* = 2, the 10 populations were divided into two groups: one comprising domesticated varieties and the other consisting of wild varieties. Notably, the Fenghua duck and mallard populations showed signs of hybridization. When *K* = 3, Pekin and Cherry Valley Pekin ducks were separated from the domesticated varieties, resulting in three populations: the shelduck population (Shaoxing ducks, Gaoyou ducks, Jinding ducks, Shanma ducks, and Jianshui yellow–brown ducks), white-feathered meat-type duck population (Pekin ducks and Cherry Valley Pekin ducks), and wild duck population (mallard, spot-billed ducks, and Fenghua ducks). However, when *K* = 4, Jianshui yellow–brown ducks were further separated from the shelduck population. The results of genetic differentiation indicated that, among the 10 populations, the highest level of genetic differentiation was observed between Jinding ducks and Gaoyou ducks (Fst = 0.217), whereas the lowest level was observed between mallard and spot-billed ducks (Fst = 0.056; Supplementary Table S5). The Jianshui yellow–brown duck population exhibited the greatest degree of differentiation from the Gaoyou duck population (Fst = 0.209) and the smallest degree of differentiation from the Fenghua duck population (Fst = 0.139).

### 3.3 Population genetic diversity

Nucleotide diversity values are indicative of the genetic diversity within a population. Supplementary Figure S2 illustrates the genetic diversity levels of the 10 populations. Mallard exhibited the highest genetic diversity, while Gaoyou ducks displayed the lowest. The two wild duck populations showed relatively rich genetic diversity. The genetic diversity of the Jianshui yellow–brown duck population was lower than that of mallard, spot-billed ducks, Shaoxing ducks, and Fenghua ducks but higher than that of Pekin ducks, Shanma ducks, Jinding ducks, Cherry Valley Pekin ducks, and Gaoyou ducks. Consequently, Jianshui yellow–brown ducks ranked fifth among the 10 populations in terms of genetic diversity.

In descending order, the values of genetic diversity were as follows: mallard, spot-billed ducks, Shaoxing ducks, Fenghua ducks, Jianshui yellow–brown ducks, Pekin ducks, Shanma ducks, Jinding ducks, Cherry Valley Pekin ducks, Gaoyou ducks. Two wild duck populations showed relatively rich genetic diversity. Among the eight domesticated populations, Jianshui yellow–brown ducks ranked third in terms of genetic diversity, after Fenghua ducks and Shaoxing ducks.

### 3.4 Selection signals

**Figure 2:**
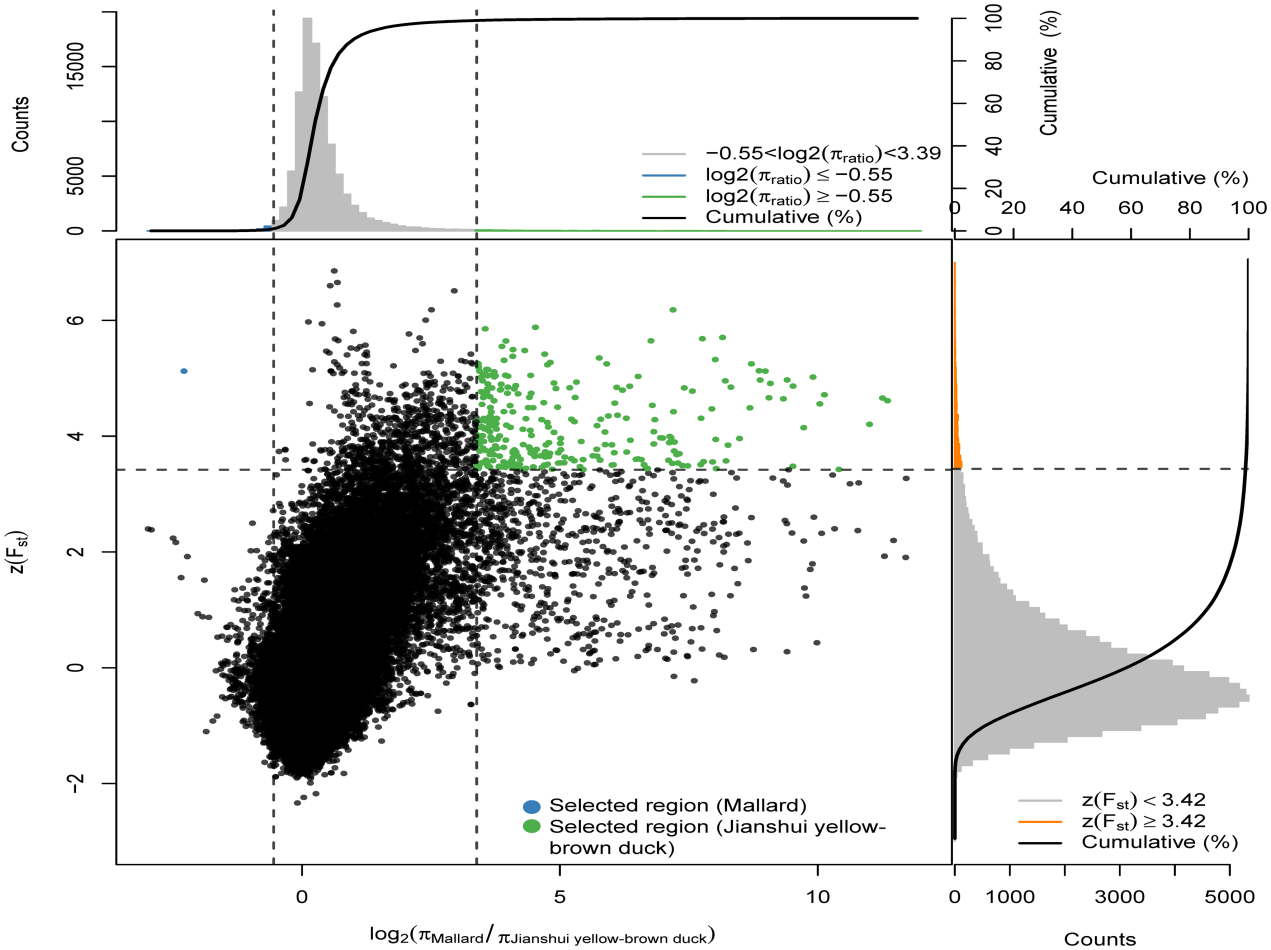
Distribution of *Z*(FST) values and log2(πMallard/Jianshui Yellow–brown duck) calculated in 40 kb sliding windows with 10 kb overlap between Jianshui yellow–brown duck and Mallard. Green dots represent selected regions on the genome of Jianshui yellow–brown duck, blue dots represent selected regions on the genome of Mallard.

Meat performance is a crucial economic trait in the domestication of ducks. To investigate the selection of functional genes related to meat traits in varieties under different degrees of selection, the selection signals of Jianshui yellow–brown ducks, which undergo low selection, and Pekin ducks, which experience high selection, were analyzed with wild mallard as the control. Fst (population fixation index) and π (nucleotide diversity) were calculated for the whole genome regions of Jianshui yellow–brown duck, Pekin duck and mallards using the sliding window method with VCFTOOLS software. The window size and step size used in the calculation were in accordance with the parameters set in the section “2.2.3 Population selection analysis.” Based on the above method, the obtained Fst values were converted into *Z*-scores, and log_2_θπ values were calculated for the compared populations. Figure 2 shows the results of the selection signals for Jianshui yellow–brown ducks and mallard. In performing the selection signal analysis, we screened genomic regions with ZFst and θπ values ranked in the top 1% as candidate regions that might be subject to selection. Specifically, we screened out regions in the genome of the Jianshui yellow–brown duck population that might be subjected to selection by setting a threshold of ZFst value >3.42 and log2(π_Mallard_/π_Jianshui yellow–brown duck_) ≥ −0.55. BEDTOOLS software was applied to merge the overlapping candidate regions, and in the results we identified 62 potential candidate genomic regions. After detailed annotation of genes within these regions, we identified a total of 136 genes that were subject to selection in Jianshui yellow–brown ducks.

**Figure 3:**
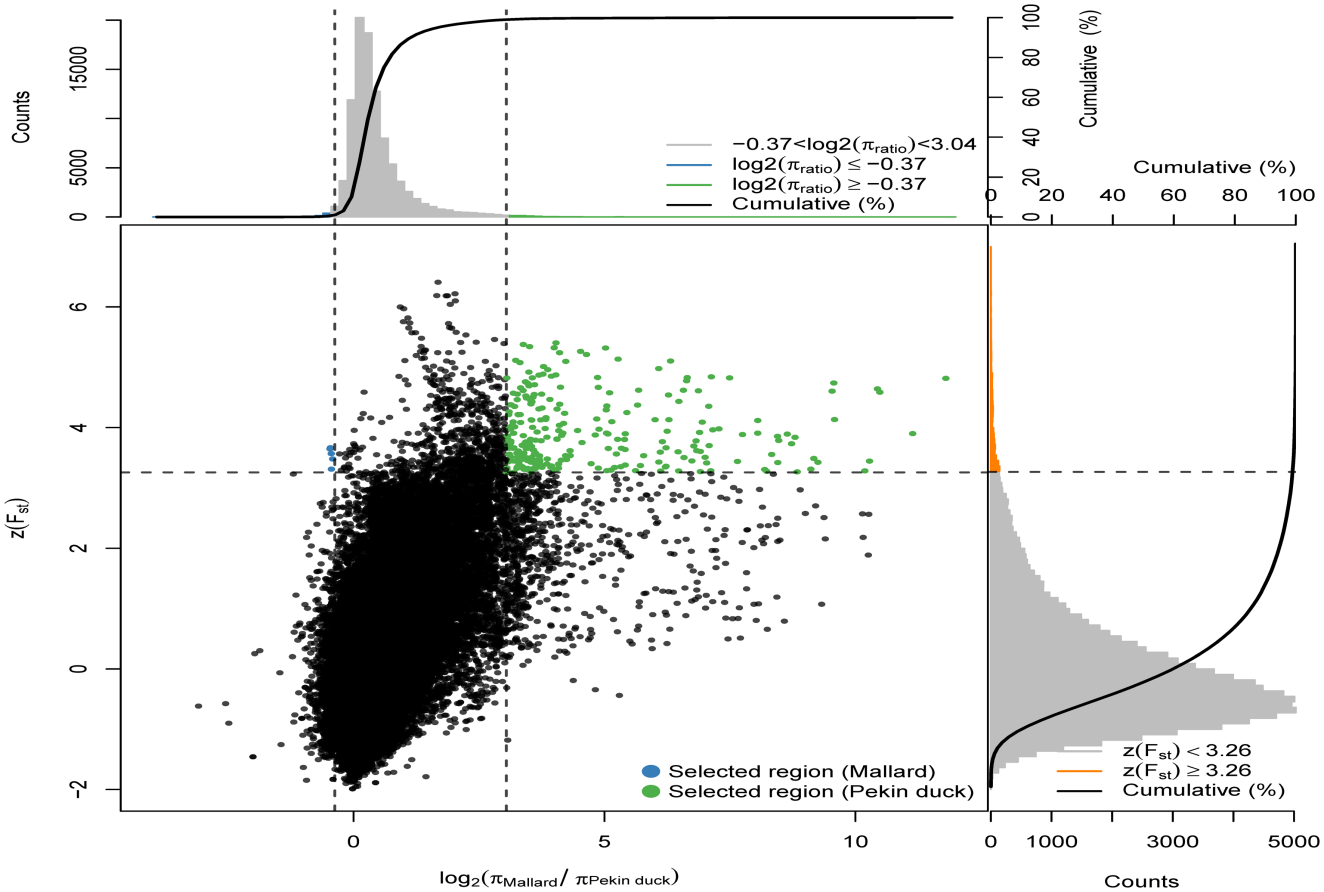
Distribution of *Z*(FST) values and log2(πMallard/rPekin duck) calculated in 40 kb sliding windows with 10 kb overlap between Pekin duck and Mallard. Green dots represent selected regions on the genome of Pekin duck, blue dots represent selected regions on the genome of Mallard.

To identify the selected genes in the Pekin duck population (Pekin duck vs. mallard), by calculating the thresholds for the ZFst and log_2_θπ values, we considered the regions with ZFst values >3.26 and log2(π_Mallard_/π_Pekin duck_) ≥ −0.37 as the regions that might be subject to selective action in the Pekin duck population (Figure 3), 90 selected genes were annotated within 63 candidate regions for Pekin ducks. Pekin ducks are globally recognized for their meat characteristics, while Jianshui yellow–brown ducks are a meat-egg-type variety with a lower degree of selective breeding. To compare the genomic differences between these two varieties, selection signal analysis was conducted on Jianshui yellow–brown ducks and Pekin ducks to identify selected genes related to meat performance in the Jianshui yellow–brown ducks population and evaluate the selective breeding of Jianshui yellow–brown ducks for meat traits. Through gene function query and Tajima’s D calculation, 6 genes associated with growth and development and lipid metabolism – *ELOVL2* (ELOVL Fatty Acid Elongase 2), *ELOVL3* (ELOVL Fatty Acid Elongase 3), *GDF10* (Growth Differentiation Factor 10), *VSTM2A* (V-Set And Transmembrane Domain Containing 2A), *PHOSPHO1* (Phosphocholine Phosphatase 1), and *IGF2BP1* (Insulin Like Growth Factor 2 MRNA Binding Protein 1) – were subjected to significant selection in the genome of Jianshui yellow–brown ducks (Supplementary Figures S3–S8). Furthermore, compared with Pekin and mallard, 6 genes related to muscle growth and fat deposition – *GDF10*, *IGF2BP1*, *SCD* (Stearoyl-CoA Desaturase), *MAP3K20* (Mitogen-Activated Protein Kinase Kinase Kinase 20), *TGFB3* (Transforming Growth Factor Beta 3), and *WNT8B* (Wnt Family Member 8B) – were identified (Supplementary Figures S9–S14). Compared with the Pekin duck population, 21 genes subjected to significant selection were observed in the Jianshui yellow–brown duck population (Supplementary Figure S15). However, based on functional annotation of these genes and comparison with the previously screened genes related to meat traits, none of these 21 genes were identified as candidate genes associated with muscle development and fat deposition.

### 3.5 Quality control of transcriptome raw data and screening of differentially expressed genes

**Figure 4:**
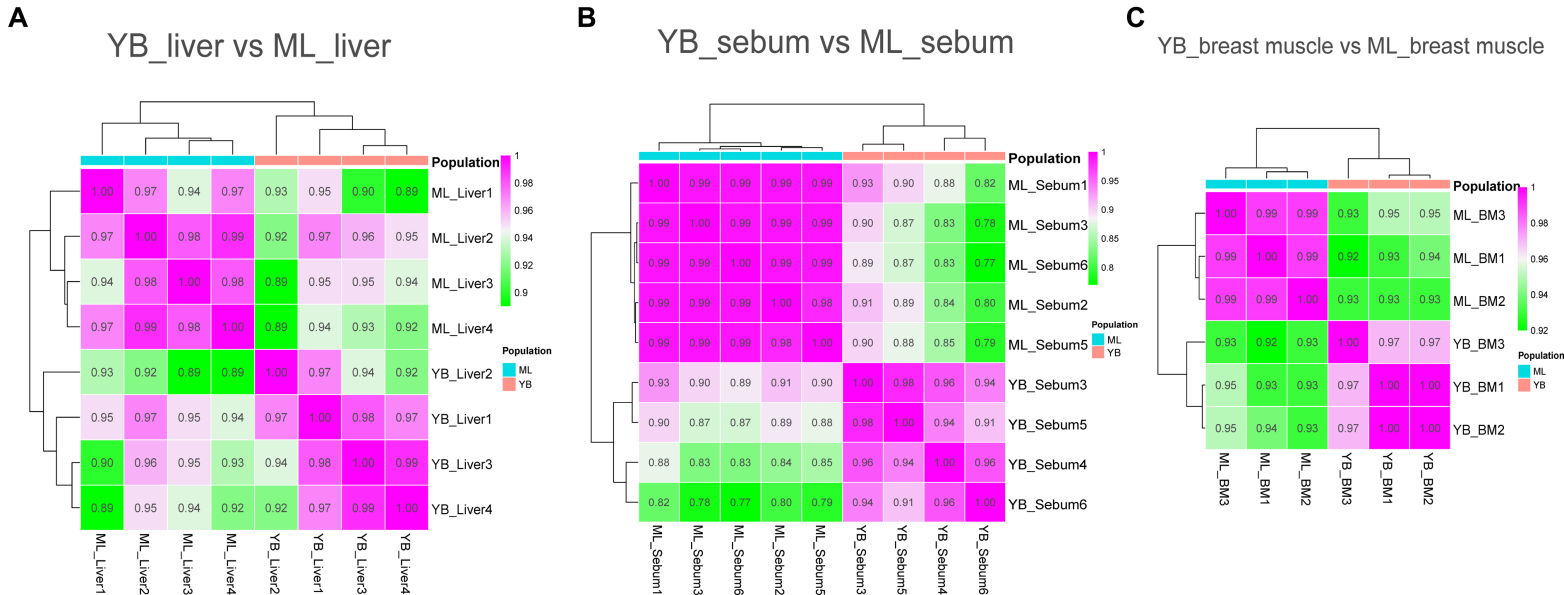
Sample correlation analysis of liver, sebum, and breast muscle transcriptome data of Jianshui yellow–brown ducks and Mallards after excluding further outlier samples. **(A)** Sample correlation thermograms of liver tissues from Jianshui yellow–brown ducks and mallards. **(B)** Sample correlation thermograms of sebum tissues from Jianshui yellow–brown ducks and Mallards. **(C)** Sample correlation thermograms of breast muscle tissues from Jianshui yellow–brown ducks and Mallards.

**Figure 5:**
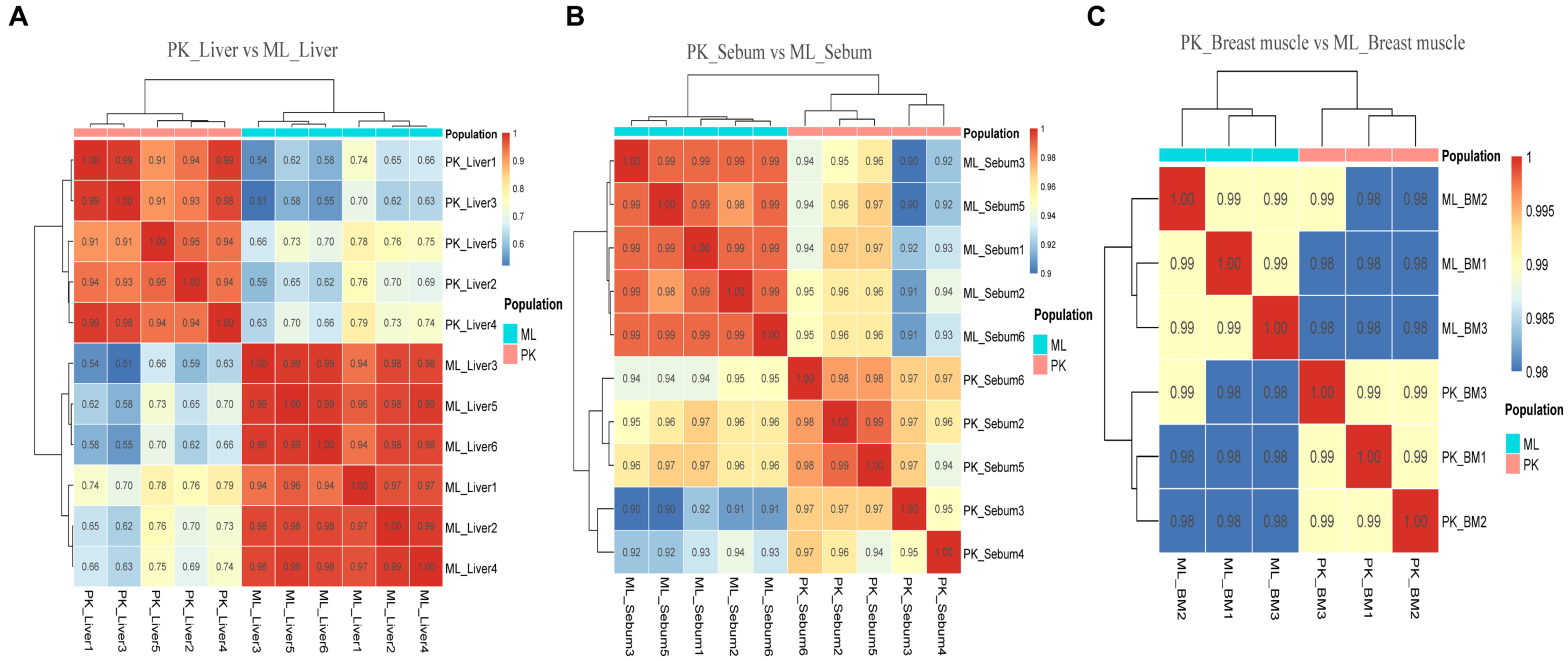
Sample correlation analysis of liver, sebum and breast muscle transcriptome data of Pekin ducks and Mallards after excluding further outlier samples. **(A)** Sample correlation thermograms of liver tissues from Pekin ducks and Mallards. **(B)** Sample correlation thermograms of sebum tissues from Pekin ducks and Mallards. **(C)** Sample correlation thermograms of breast muscle tissues from Pekin ducks and Mallards.

Transcriptome sequencing technology facilitates the detection of mRNA expression in the same tissue across different duck varieties. This allows for the annotation and enrichment analysis of the functions of Differently Expressed genes (DEGs), which facilitates the identification of important functional genes related to characteristic traits in various varieties. In this study, transcriptome sequencing analysis was conducted on the liver, sebum and breast muscle tissues of Jianshui yellow–brown ducks and mallards. Additionally, DEGs were screened in the liver, sebum and breast muscle tissues between Pekin ducks and mallards. After screening and quality control of the raw data of transcriptome sequencing, and then comparing the high-quality reads back to the reference genome of ducks (CAU_duck1.0: https://ftp.ensembl.org/pub/release-110/fasta/anas_platyrhynchos_platyrhynchos/dna/Anas_platyrhynchos_platyrhynchos.CAU_duck1.0.dna.toplevel.fa.gz), the results showed that the transcriptome sequencing results of the tissue samples from the Jianshui yellow and brown duck population were of good quality, with an average Q20, Q30, and comparison rate of 92.61, 97.11, and 78.92%, respectively (Supplementary Table S6). The transcriptome sequencing results of Pekin duck and mallard duck downloaded from public databases also showed a high quality level, with average Q20, Q30 and alignment rates of 94.17, 97.48, and 81.01%, respectively (Supplementary Table S7). After quantification of gene expression, sample correlation analysis was performed on the normalized data using the TPM correction method. Subsequently, sequencing quality and sample data quality were preliminarily examined through analyses of sequencing base quality values, alignment rates, and sample correlation. Sample correlation analysis was performed with six sets of transcriptome data obtained from the same tissue among different duck varieties. The results revealed the presence of abnormal data (Supplementary Figures S16–S17). To improve the credibility of the results, sample correlation analysis was repeated after removing the abnormal data, and the results of this analysis suggested that the intra-population correlation was higher than the inter-population correlation, and samples from the same variety could be clustered together (Figures 4, 5). Samples with high confidence were then used to screen for DEGs, using threshold values of |log2FoldChange| > 1.5 and Padj < 0.001. According to the presentation in Supplementary Figures S18A–C, the transcriptomic data of Jianshui yellow–brown ducks and mallards showed that these two breeds exhibited different gene expression profiles in liver, sebum and breast muscle tissues. Specifically, there were 464 differentially expressed genes in liver tissues (251 up-regulated and 213 down-regulated), 1,435 differentially expressed genes in sebum tissues (958 up-regulated and 477 down-regulated), and 1,290 differentially expressed genes in breast muscle tissues (439 up-regulated and 851 down-regulated genes). After another transcriptome analysis of Pekin and mallard duck tissues (Supplementary Figures S18D–F), we identified 2,050 differentially expressed genes in liver tissues (532 genes were up-regulated and 1,518 genes were down-regulated), 473 differentially expressed genes in subcutaneous adipose tissues (including 295 genes up-regulated and 178 genes down-regulated), and 475 differentially expressed genes were screened out of breast muscle tissues (185 genes were up-regulated and 290 genes were down-regulated), differentially expressed genes between groups can be viewed in Supplementary Tables S8–S13.

### 3.6 Joint analysis results

**Figure 6:**
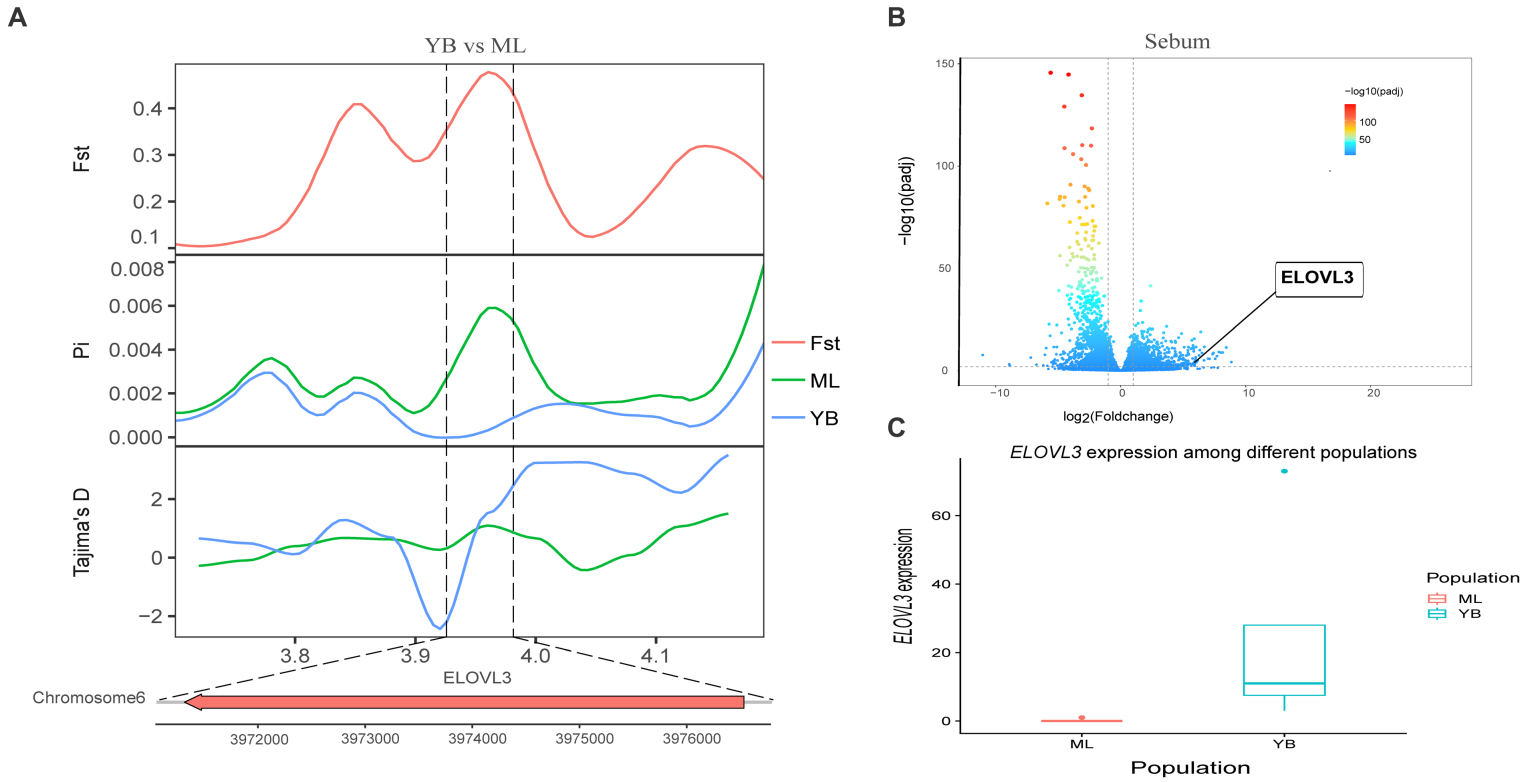
Combined analysis of genomic selection signal candidate genes and transcriptome differentially expressed genes in Jianshui yellow–brown duck and Mallard. **(A)** Fst Pi and Tajima’s D value of *ELOVL3* between Jianshui Yellow–brown duck and Mallard. **(B)** Differentially expressed genes in sebum of Jianshui yellow–brown and Mallard. **(C)** Gene expression of *ELOVL3* in sebum tissue of Jianshui yellow–brown ducks and Mallards.

**Figure 7:**
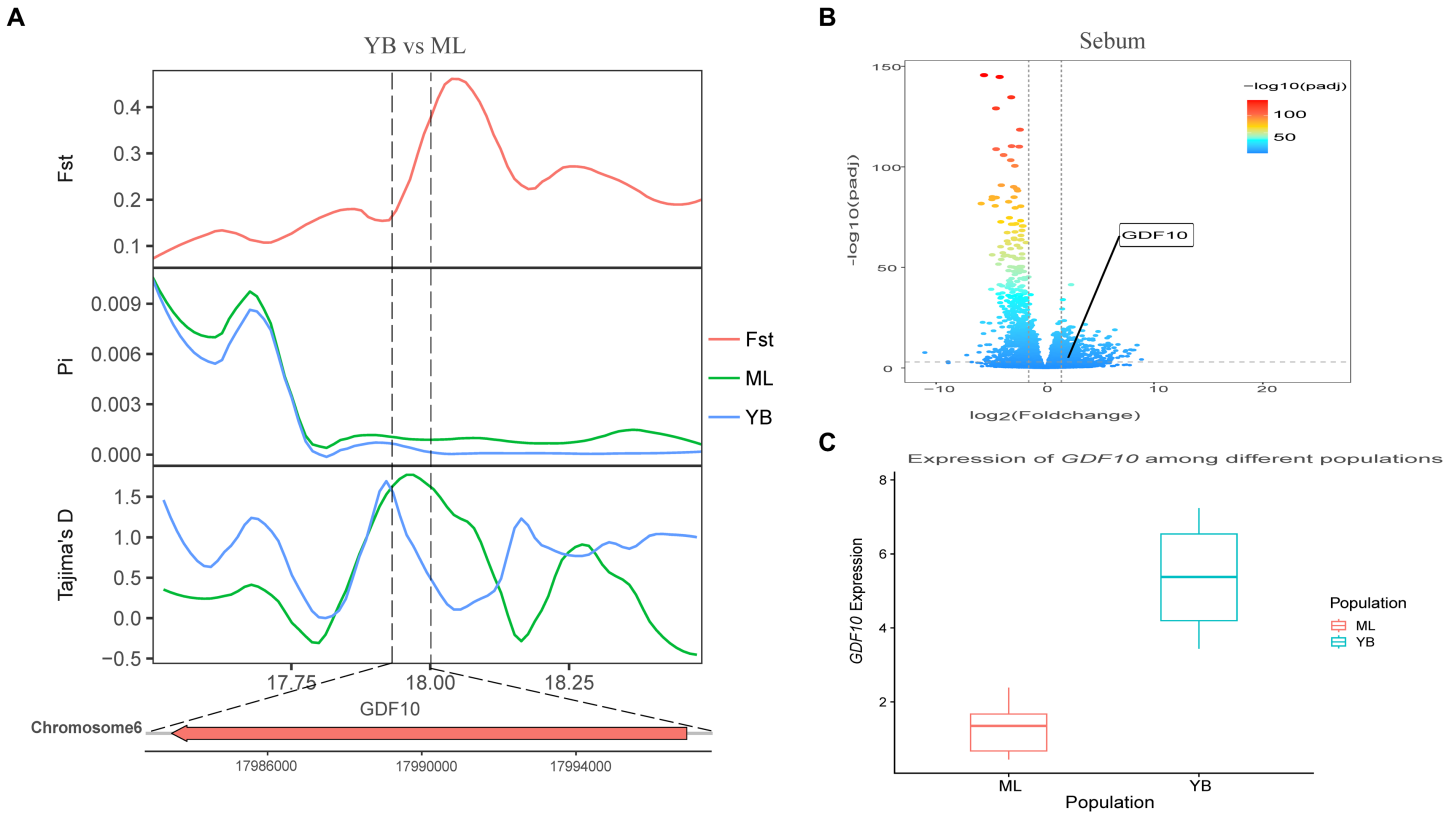
Combined analysis of genomic selection signal candidate genes and transcriptome differentially expressed genes in Jianshui yellow–brown duck and Mallard. **(A)** Fst Pi and Tajima’s D value of *GDF10* between Jianshui Yellow–brown duck and Mallard. **(B)** Differentially expressed genes in sebum of Jianshui yellow–brown and Mallard. **(C)** Gene expression of *GDF10* in sebum tissue of Jianshui yellow–brown ducks and Mallards.

**Figure 8:**
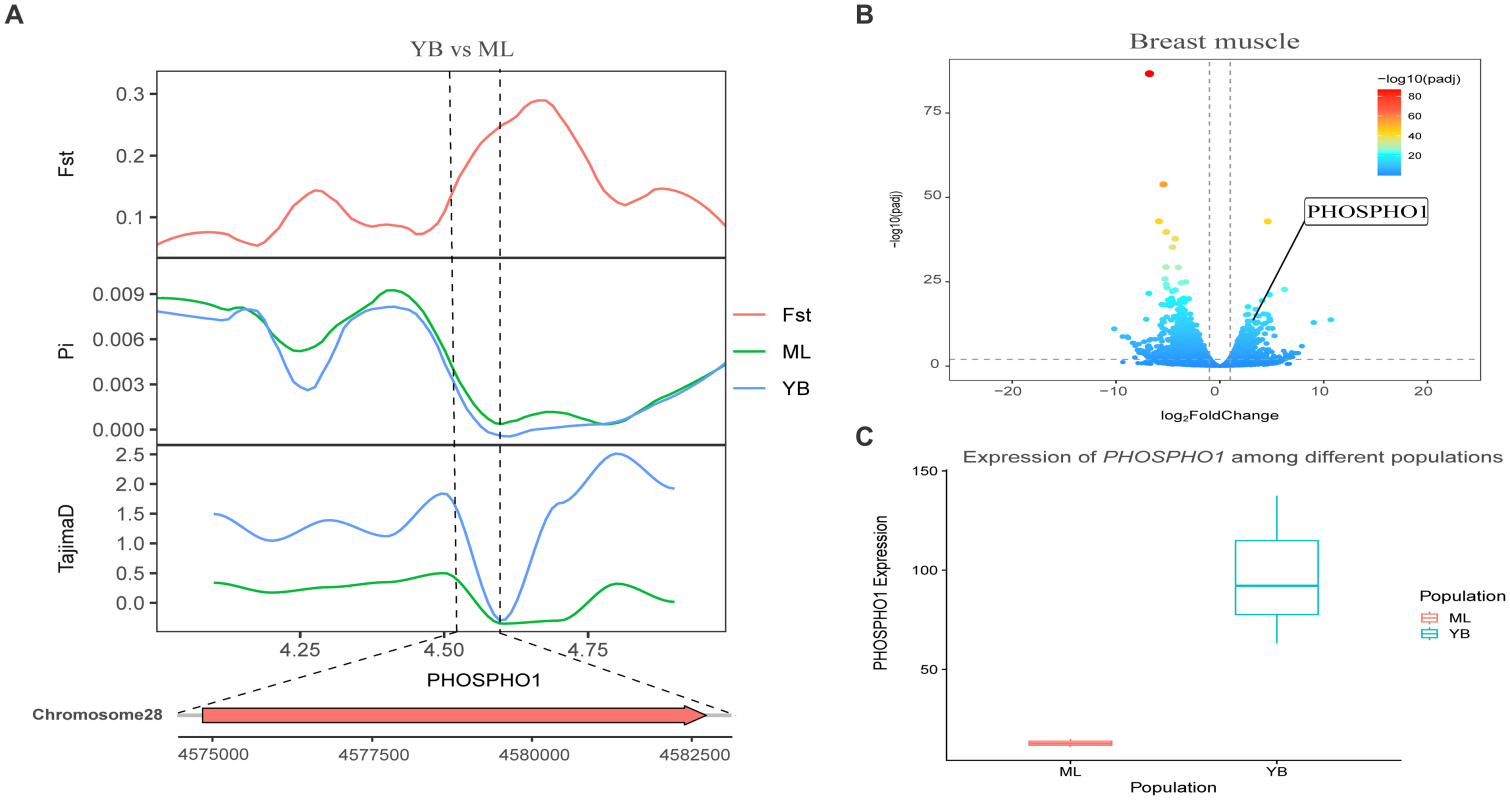
Combined analysis of genomic selection signal candidate genes and transcriptome differentially expressed genes in Jianshui yellow–brown duck and Mallard. **(A)** Fst Pi and Tajima’s D value of *PHOSPHOI* between Jianshui Yellow–brown duck and Mallard. **(B)** Differentially expressed genes in breast muscle of Jianshui yellow–brown and Mallard. **(C)** Gene expression of *PHOSPHOI* in breast muscle tissue of Jianshui yellow–brown ducks and Mallards.

**Figure 9:**
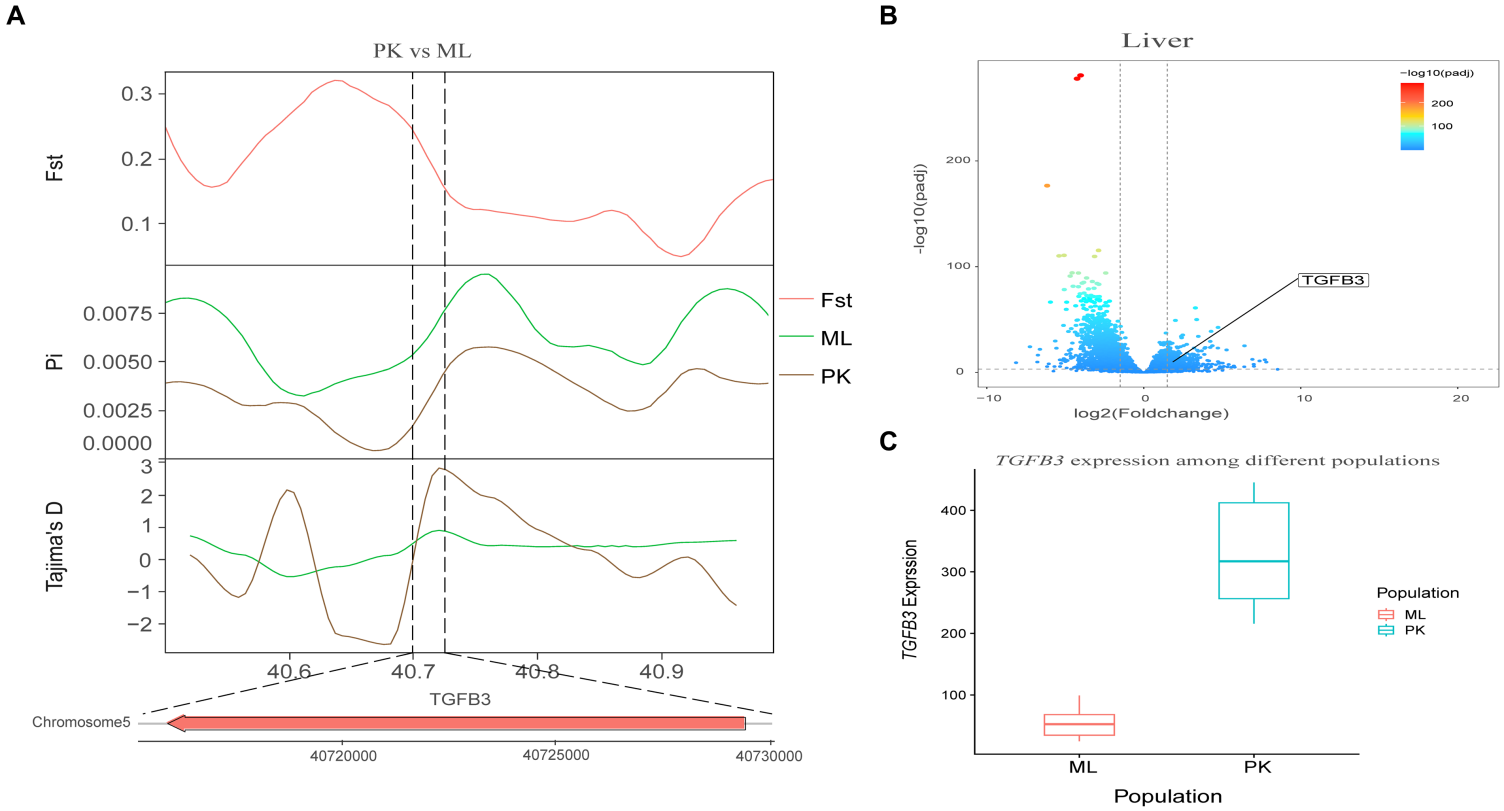
Combined analysis of genomic selection signal candidate genes and transcriptome differentially expressed genes in Pekin duck and Mallard. **(A)** Fst Pi and Tajima’s D value of *TGFB3* between Pekin duck and Mallard. **(B)** Differentially expressed genes in liver of Pekin duck and Mallard. **(C)** Gene expression of *TGFB3* in liver tissue of Pekin ducks and Mallards.

**Figure 10:**
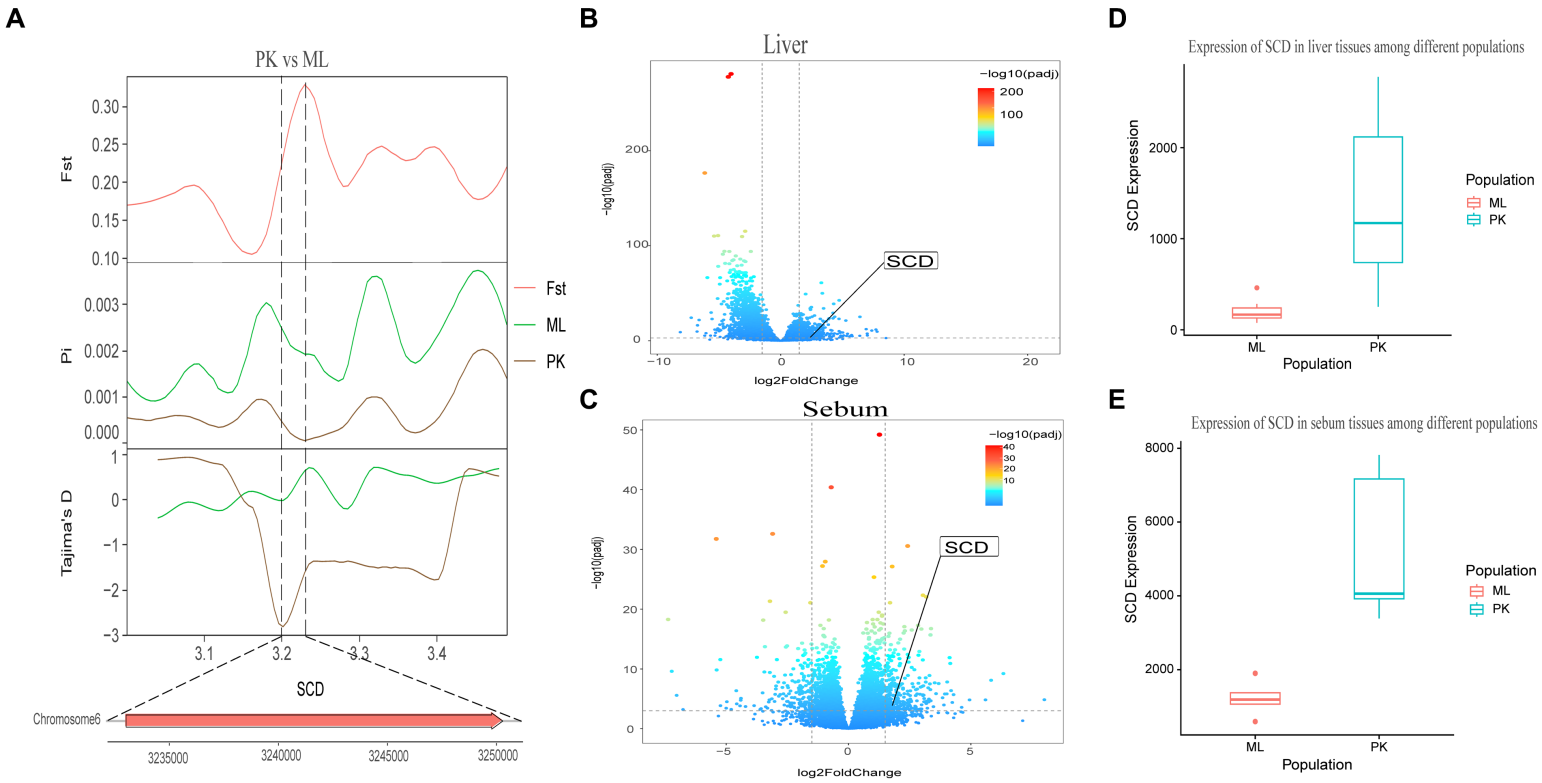
Combined analysis of genomic selection signal candidate genes and transcriptome differentially expressed genes in Pekin duck and Jianshui yellow–brown duck. **(A)** Fst Pi and Tajima’s D value of *SCD* between Pekin duck and Mallard. **(B)** Differentially expressed genes in liver of Pekin duck and Mallard. **(C)** Differentially expressed genes in sebum of Pekin duck and Mallard. **(D)** Gene expression of *SCD* in liver tissue of Pekin ducks and Mallards. **(E)** Gene expression of *SCD* in sebum tissue of Pekin ducks and Mallards.

Genotypic differences between populations within the region under selection may trigger changes in the expression of the gene. Multi-omics data were utilized to identify reliable artificial selection loci and functional genes associated with economically important traits. Five functional genes (*ELOVL2*, *ELOVL3*, *GDF10*, *VSTM2A*, and *IGF2BP1*) that may affect meat production and quality in Jianshui yellow–brown ducks were identified from genome-wide signal selection analysis. Transcriptome analysis of DEGs revealed significant differences in *PHOSPHO1* gene expression in the breast muscle tissues of Jianshui yellow–brown ducks and mallards (Figure 6). In addition, the expression levels of *ELOVL3* and *GDF10* genes also showed significant differences in sebum tissue (Figures 7, 8). To identify the functional genes related to muscle growth and fat deposition traits in Pekin ducks, and provide references for the selective breeding of meat-type duck varieties, genomic selection signal detection was performed on Pekin ducks and mallards. This analysis led to the identification of six functional genes related to meat traits (*GDF10*, *IGF2BP1*, *SCD*, *MAP3K20*, *TGFB3*, and *WNT8B*). Data on DEGs indicated significant differences in *TGFB3* gene expression in the liver tissue and *SCD* gene expression in both liver and sebum tissues (Figures 9, 10).

## 4 Discussion

### 4.1 Molecular genetic characterization of Jianshui yellow–brown ducks

To comprehensively and accurately assess the germplasm resources of Jianshui yellow–brown ducks, resequencing was performed on 18 Jianshui yellow–brown ducks. The resequencing data from the ducks in the NCBI database were also used for population structure analysis. PCA was performed based on genome-wide SNPs. The results revealed that the Jianshui yellow–brown duck population was clustered into a single category, and individuals of the same species could be clustered together without any outlier samples in the PCA plot. In the evolutionary tree, 110 individuals from 10 populations were categorized into four groups. Among them, Shaoxing ducks, Gaoyou ducks, Shanma ducks, and Jinding ducks were clustered into one category, and all four groups were shelduck varieties, originating from East China. Thus, they were implicated to have a closer genetic relationship than the other six populations. Mallards, spot-billed ducks, and Fenghua ducks were clustered into a single category. Although differences in body size and appearance existed between mallards and spot-billed ducks, the degree of genetic differentiation between the two varieties was small. Therefore, no reproductive isolation occurred after mating, and separating the two populations in the evolutionary tree was challenging (5, 24). In the relationship tree, Fenghua ducks and mallards showed a closer genetic relationship. Fenghua ducks are directly domesticated from mallard ducks. Because of the short period of domestication, Fenghua ducks still retain some of the habits and characteristics of mallards (4, 25). Two white-feathered varieties, namely, Pekin duck and Cherry Valley Pekin duck, were clustered into a single category owing to their close genetic relationship. The Cherry Valley Pekin duck is a duck variety known for its high-quality meat, and this variety was improved and bred on the Chinese native Pekin duck by the British Cherry Valley Company. Therefore, the Cherry Valley Pekin duck and the Pekin duck exhibited a close genetic relationship (3, 26). In addition, the Jianshui yellow–brown duck population was clustered into a separate category, a finding similar to those of the distribution of the Jianshui yellow–brown duck population in PCA. Compared with PCA and evolutionary tree analysis, structure analysis can be used to determine the reasonableness of grouping and gene exchange among different varieties. In structure analysis, when the cross-validation error rate was minimum (i.e., *K* = 4), 10 populations were categorized into four groups. Jianshui yellow–brown ducks were separated from the shelduck population, forming a distinct population. This finding validated the results from PCA and evolutionary tree analysis, highlighting the differences in molecular genetic characteristics between Jianshui yellow–brown ducks and wild ducks, white-feathered meat-type ducks, and shelducks in East China. We hypothesized that the Jianshui yellow–brown ducks have developed a unique molecular genetic mechanism owing to the influence of natural and artificial selection.

### 4.2 Selection signal analysis of Jianshui yellow–brown ducks, Pekin ducks, and mallard

Jianshui yellow–brown ducks, known for their tender meat, have become the key ingredient for the specialty dish “Qujiang Roast Duck” in Jianshui County, Yunnan Province, China. Identifying candidate genes that affect the traits of these varieties is crucial for understanding the genetic basis of their characteristics and conserving germplasm resources. In the selective breeding process, selection signal analysis was conducted on Jianshui yellow–brown ducks and mallard to explore the functional genes influencing meat production and meat quality traits. Several candidate genes related to muscle quality were identified, including members of the elongase of the very-long-chain fatty acid (ELOVL) gene family, such as *ELOVL2* and *ELOVL3*, both of which were subjected to significant selection. The ELOVL family, as initiation and rate-limiting enzymes that catalyze fatty acid synthesis, plays an important role in fatty acid chain elongation (27). *VSTM2A* plays an essential role in regulating preadipocyte differentiation. *VSTM2A* overexpression increased the efficiency of fat synthesis, whereas *VSTM2A* knockdown inhibited fat formation (28, 29). Growth-related genes, including *GDF10*, *IGF2BP1*, and *PHOSPHO1* were also identified. Gene polymorphisms in *GDF10*, also known as bone morphogenetic protein-3b (BMP-3b), have shown a significant correlation with some growth traits in cattle and have a possible involvement in skeletal muscle formation and development. The *GDF10* gene is also involved in lipogenesis and metabolism (30). Based on the results of GWAS analysis, Zhou et al. identified *IGF2BP1* as a candidate gene affecting the growth and carcass of domestic ducks (20). Moreover, the study by Wang et al. on domestic chickens, which was based on the pan-genomic data of domestic ckickens, showed that high expression of the *IGF2BP1* gene influences the body size of ckickens (31). The *PHOSPHO1* is a member of the phosphate dehydrogenase family and encodes an enzyme belonging to the acid phosphatase class that catalyzes the hydrolysis of inorganic phosphate esters. This gene is well known for its key role in bone growth and bone mineralization in organisms (32, 33). Recently, experimental results from Peng et al. have also shown that the *PHOSPHO1* gene also plays an important role in the regulation of myogenic cell differentiation (34). These findings contribute to understanding the genetic mechanisms underlying the meat production and quality characteristics of Jianshui yellow–brown ducks.

Based on the analysis of population structure, it was found that the Jianshui yellow–brown duck and Pekin duck, two domesticated varieties, have significantly diverged from the wild mallard through domestication and selective breeding. In the selection signal analysis between the Jianshui yellow–brown duck and mallard, candidate genes related to meat traits of the Jianshui yellow–brown duck were identified. However, as a meat-egg-type local variety, the meat performance of Jianshui yellow–brown ducks still requires further improvement. Therefore, the meat-type Pekin duck was chosen as the research object and the mallard as the control, and the selection signaling was used to identify candidate genes affecting muscle growth and fat deposition traits in Pekin duck. Six functional genes (*GDF10*, *IGF2BP1*, *SCD*, *MAP3K20*, *TGFB3*, and *WNT8B*) related to meat traits were identified. Among them, *SCD*, a key enzyme required for the synthesis of monounsaturated fatty acids, plays a crucial role in the regulation of lipogenesis. Mice with *SCD1* gene knockout have increased energy expenditure and fat consumption and decreased amounts of fat deposition (35). Bioinformatics approaches have been used for studying meat quality in livestock, and the results have suggested that the *SCD* gene is an important functional gene affecting lipogenesis and fat deposition (36, 37). *TGFB3*, a candidate gene affecting variation in rib count in pigs, also plays a role in avian lipogenesis and lipometabolism. Based on the GWAS-derived SNP association results affecting abdominal fat percentage in chickens, *TGFB3* was identified as a candidate functional gene affecting fat deposition (38). Wang et al. identified *TGFB3* as a target gene of miR-122, which is a regulator of adipose metabolism (39, 40). *MAP3K20* and *WNT8B*, belonging to the MAPK and Wnt signaling pathways (41, 42), respectively, are involved in cellular signaling pathways crucial for muscle growth and development. These genes may have a regulatory role in the muscle growth of Pekin ducks.

Jianshui yellow–brown duck, as a meat-egg-type local variety, has undergone limited selective breeding for meat traits. In contrast, the Pekin duck is a meat-type variety undergoing extensive selective breeding. To explore the genetic differences between these two varieties and assess the selection potential of Jianshui yellow–brown duck for meat performance, a selection signal analysis was performed. Only 21 genes were subjected to selection in the Jianshui yellow–brown duck population compared with those in the Pekin duck. When using mallard as a control, 136 and 90 genes were selected in the Jianshui yellow–brown duck and Pekin duck populations, respectively. It is worth noting that during the functional annotation of these 21 genes (YB vs. PK) selected from the Jianshui yellow–brown duck population, we also found an interesting gene, *ITGA4* (Integrin Subunit Alpha 4), which encodes the α4 integrin, which has been shown to play an important role in the regulation of inflammation and immune responses (43, 44). Jianshui yellow–brown ducks have always lived at relatively high altitudes and, due to their relatively short history of domestication, they exhibit strong environmental adaptations and disease resistance. Compared with the Pekin duck population, we observed that the *ITGA4* was strongly selected for in the genome, and hypothesized that the *ITGA4* plays an important role in both environmental adaptation and disease resistance in Jianshui yellow–brown ducks.

Among these selected genes, 6 genes were associated with muscle growth and fat deposition in Jianshui yellow–brown ducks, and 6 genes were related to meat traits in Pekin ducks. However, these genes were not detected in the Jianshui yellow–brown duck population when comparing selection signals between Jianshui yellow–brown ducks and Pekin ducks. Although the Jianshui yellow–brown duck variety has been bred for its meat characteristics for some time, the degree of its improvement in meat traits was not adequately high because of the short duration and low intensity of selective breeding. Therefore, further selective breeding efforts are needed to enhance meat yield and quality in Jianshui yellow–brown ducks.

### 4.3 Joint analysis of genomic selection signals and transcriptome

The selection signaling method has gained attention for identifying important functional genes related to economic traits in ducks (6, 45–47). By screening genes within regions subject to selection, which exhibit reduced polymorphism, higher inter-population differentiation, and changes in allele frequency, the accuracy of results can be improved (48). Combining multiple selection signal assays enhances the reliability of the findings. Additionally, the availability of upgraded reference genomes facilitates gene annotation within selected regions (49–51). Transcription of DNA into mRNA is a key event for proteins to exert their functions. However, gene expression during the transcription process is spatiotemporal specific, wherein different growth periods and feeding conditions affect the degree of gene expression (52, 53). Therefore, joint analysis of selection signals and transcriptome data provides a preliminary understanding of the effect of allelic changes in the selected region on gene expression and facilitates the identification of reliable selection sites and functional genes (1, 20). In this study, *ELOVL3*, *GDF10*, and *PHOSPHO1* were identified as important functional genes affecting muscle growth and fat deposition in Jianshui yellow–brown ducks through a joint analysis of selection signals and transcriptome data. Moreover, the selection signal analysis between Jianshui yellow–brown ducks and Pekin ducks revealed that genes related to meat traits in the Jianshui yellow–brown duck population were not strongly selected, indicating the need for further emphasis on selective breeding for meat traits. The *TGFB3* and *SCD* genes identified in Pekin ducks, associated with meat performance, may serve as potential markers for the molecular breeding of meat-type Jianshui yellow–brown ducks in the future.

## 5 Conclusion

The analysis of genetic structure based on whole-genome resequencing data revealed that the Jianshui yellow–brown duck represents a distinct population category with unique genetic mechanisms, possibly attributed to natural adaptation and artificial breeding. The integration of selection signaling and transcriptomic methods led to the identification of *ELOVL3*, *GDF10*, and *PHOSPHO1* as important functional genes influencing meat traits in Jianshui yellow–brown ducks. Compared with the selection signals in Pekin duck, Jianshui yellow–brown ducks have undergone less intense selective breeding for meat performance, necessitating further efforts to enhance their meat yield and quality. These findings provide valuable insights for the conservation and utilization of Jianshui yellow–brown duck germplasm resources and guide future selective breeding endeavors.

## Data availability statement

The original contributions presented in the study are publicly available. This data can be found here: National Center for Biotechnology Information (NCBI) BioProject, https://www.ncbi.nlm.nih.gov/bioproject/, PRJNA1000221 and PRJNA1000222.

## Ethics statement

The animal study was approved by Yunnan Agricultural University Laboratory Animal Welfare Ethics Committee; Acceptance No. 202103035. The study was conducted in accordance with the local legislation and institutional requirements.

## Author contributions

XL: Formal analysis, Software, Visualization, Writing – original draft. AX: Supervision, Project administration, Writing – original draft. LM: Investigation, Software, Writing – original draft. XG: Conceptualization, Writing – original draft. SF: Methodology, Writing – original draft. XD: Methodology, Writing – original draft. BN: Software, Writing – original draft. LT: Software, Writing – original draft. LZ: Software, Writing – original draft. DY: Conceptualization, Funding acquisition, Supervision, Writing – review & editing. XK: Funding acquisition, Project administration, Supervision, Writing – review & editing.
