# Supplementary material for: Molecular genetic characterization and meat-use functional gene identification in Jianshui yellow–brown ducks through combined resequencing and transcriptome analysis
Source: Front Vet Sci. 2023 Dec 12;10:1269904. doi: 10.3389/fvets.2023.1269904 (PMC10765987; doi:10.3389/fvets.2023.1269904)
Supplement: Supplementary file 1 [file Data_Sheet_1.zip › Supplementary Figures.docx]

Supplementary Figure


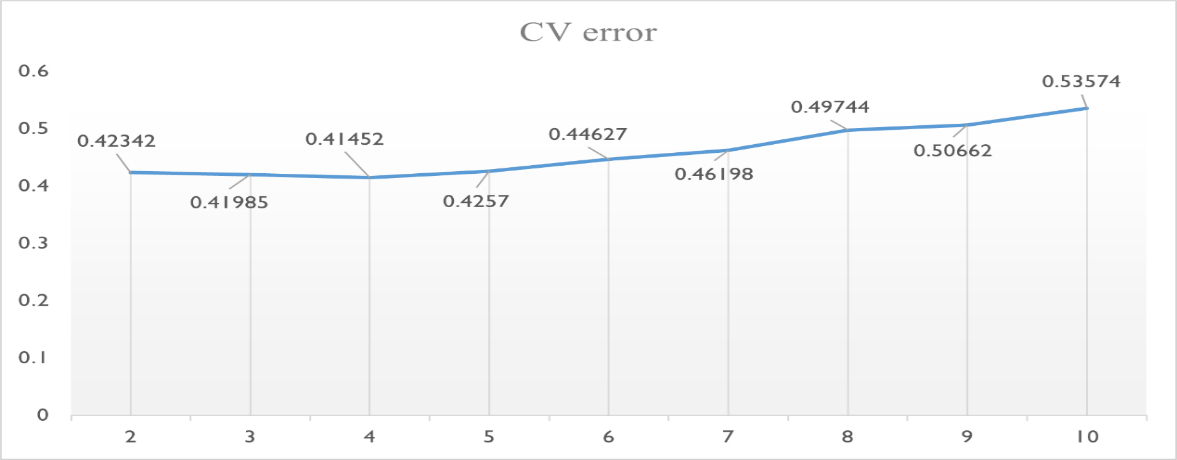


**Figure S1.** Cross validation error.


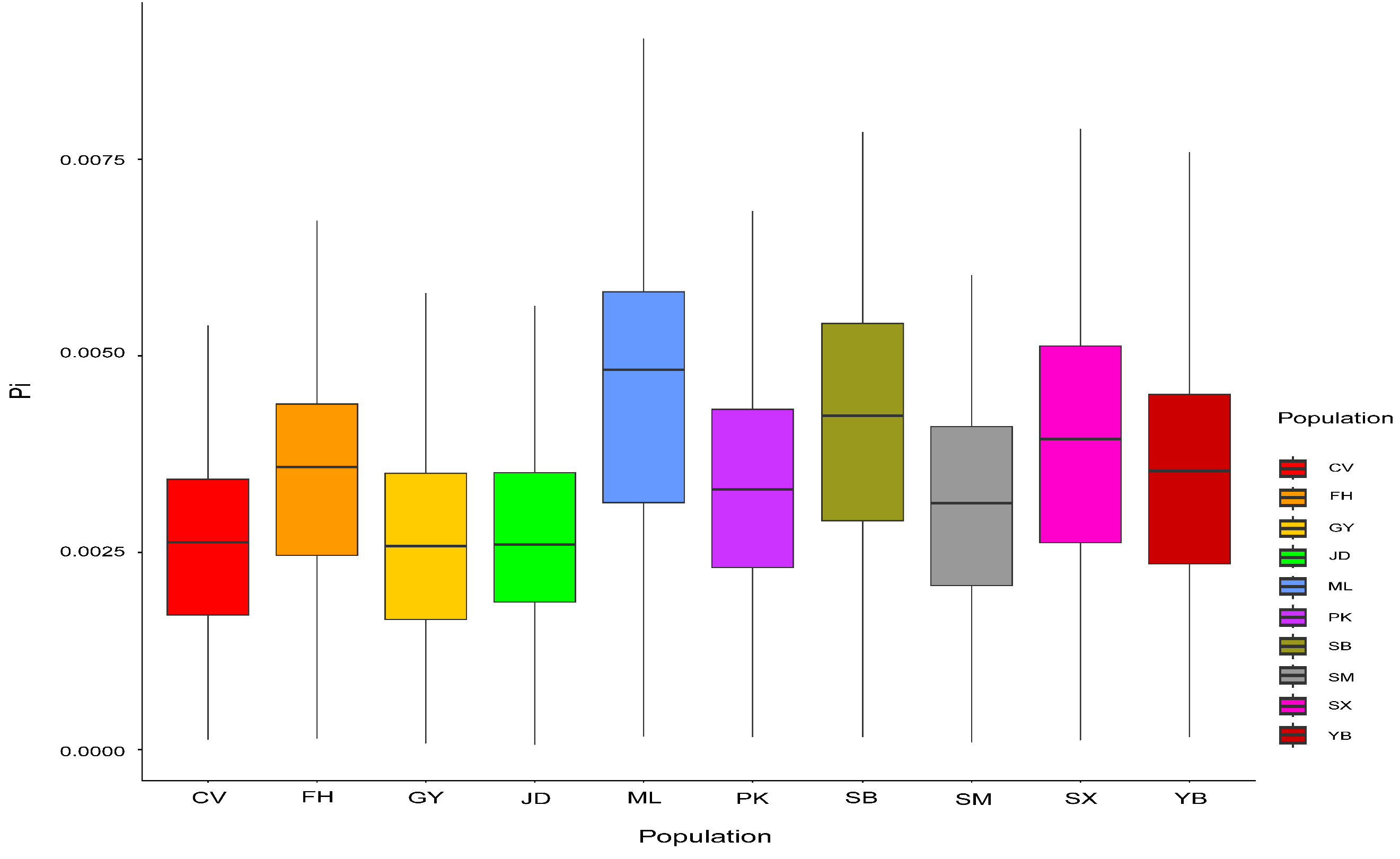


**Figure S2.** Population nucleotide diversity(Cherry Valley Pekin duck(CV), Fenghua duck(FH) , Gaoyou duck(GY), Jinding duck(JD), Mallard(ML), Pekin duck(PK), Spot-billed duck(SB), Shanma duck(SM), Shaoxing duck(SX), Jianshui Yellow-brown duck(YB)). The x-axis represents the duck population, and the y-axis represents the nucleotide diversity(Pi) of the population.


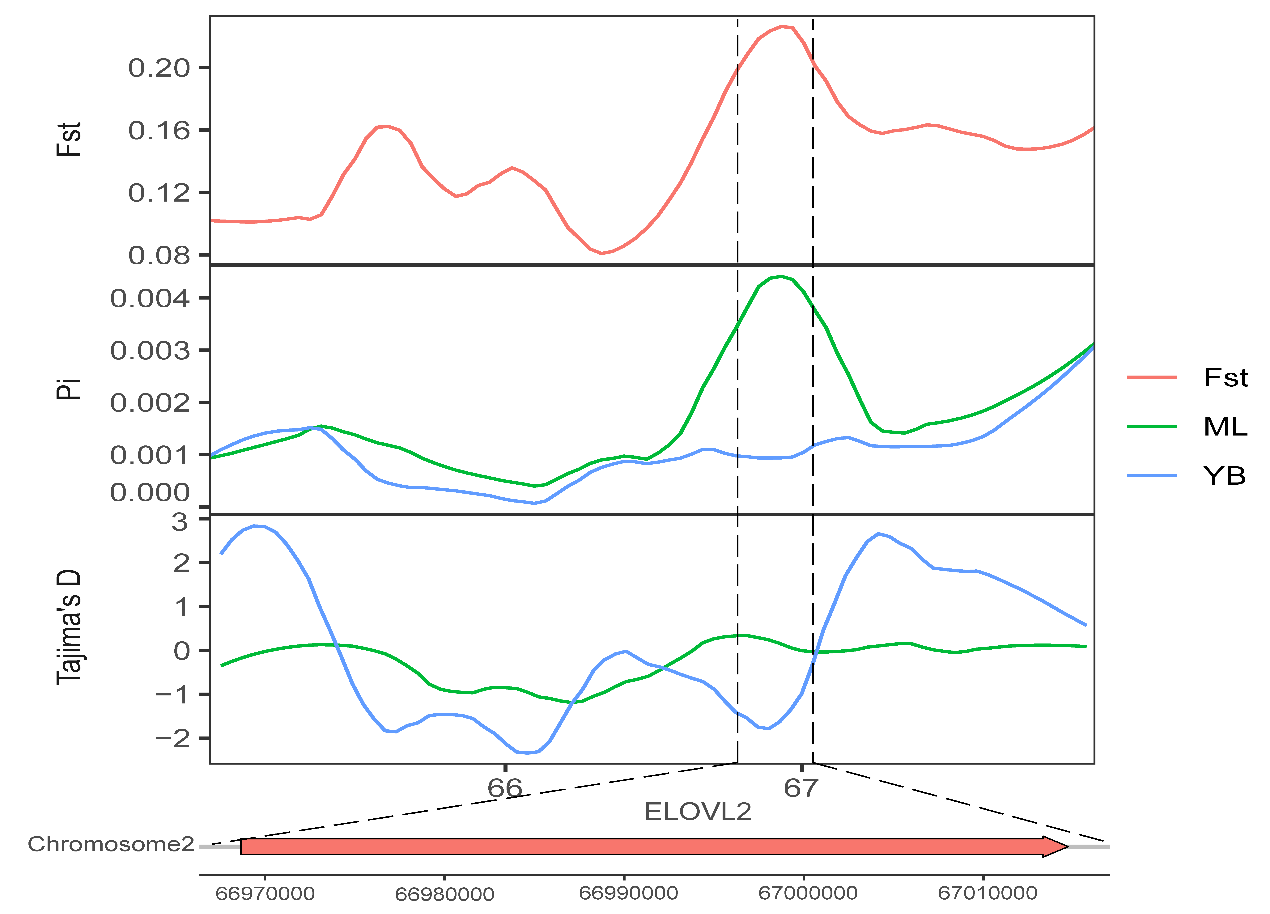


**Figure S3.** Fst、Pi and Tajima’s D value of *ELOVL2* between Jianshui Yellow-brown duck and Mallard.


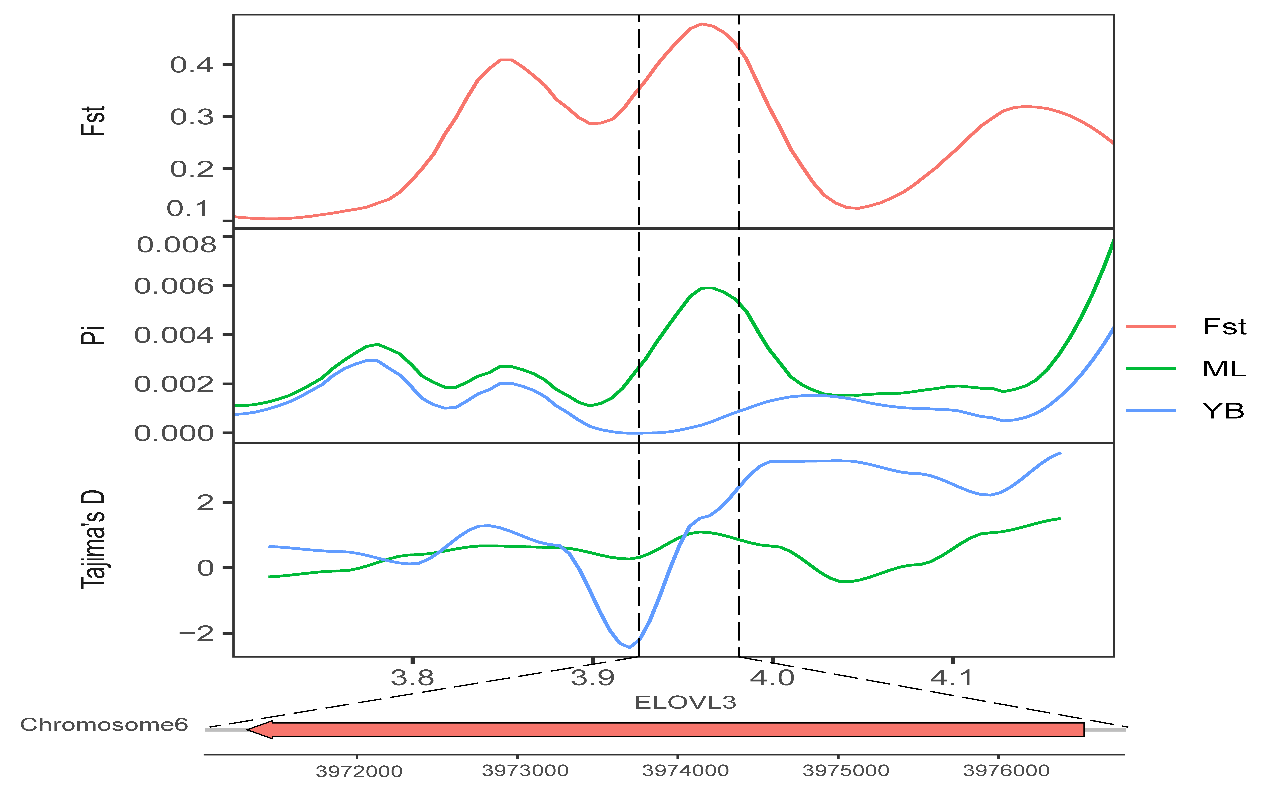


**Figure S4.** Fst、Pi and Tajima’s D value of *ELOVL3* between Jianshui Yellow-brown duck and Mallard.


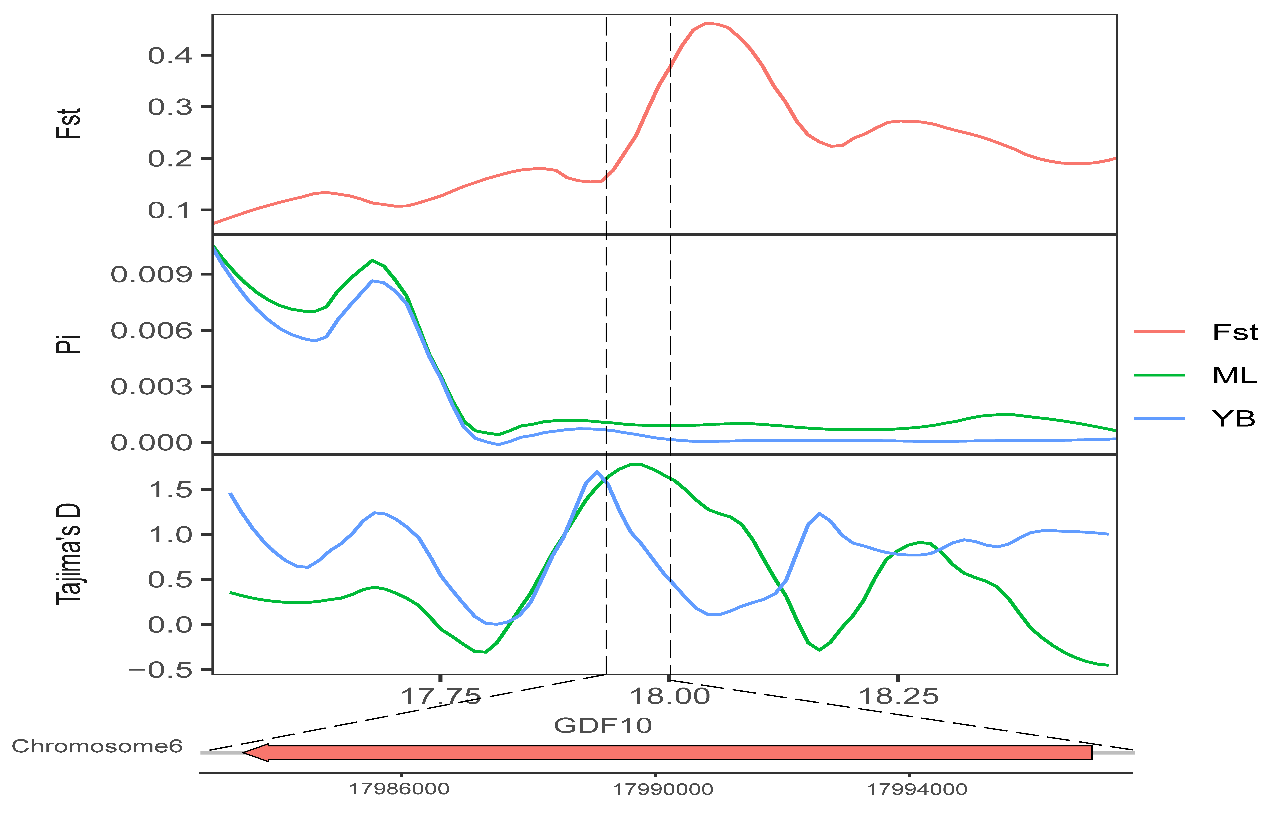


**Figure S5.** Fst、Pi and Tajima’s D value of *GDF10* between Jianshui Yellow-brown duck and Mallard.


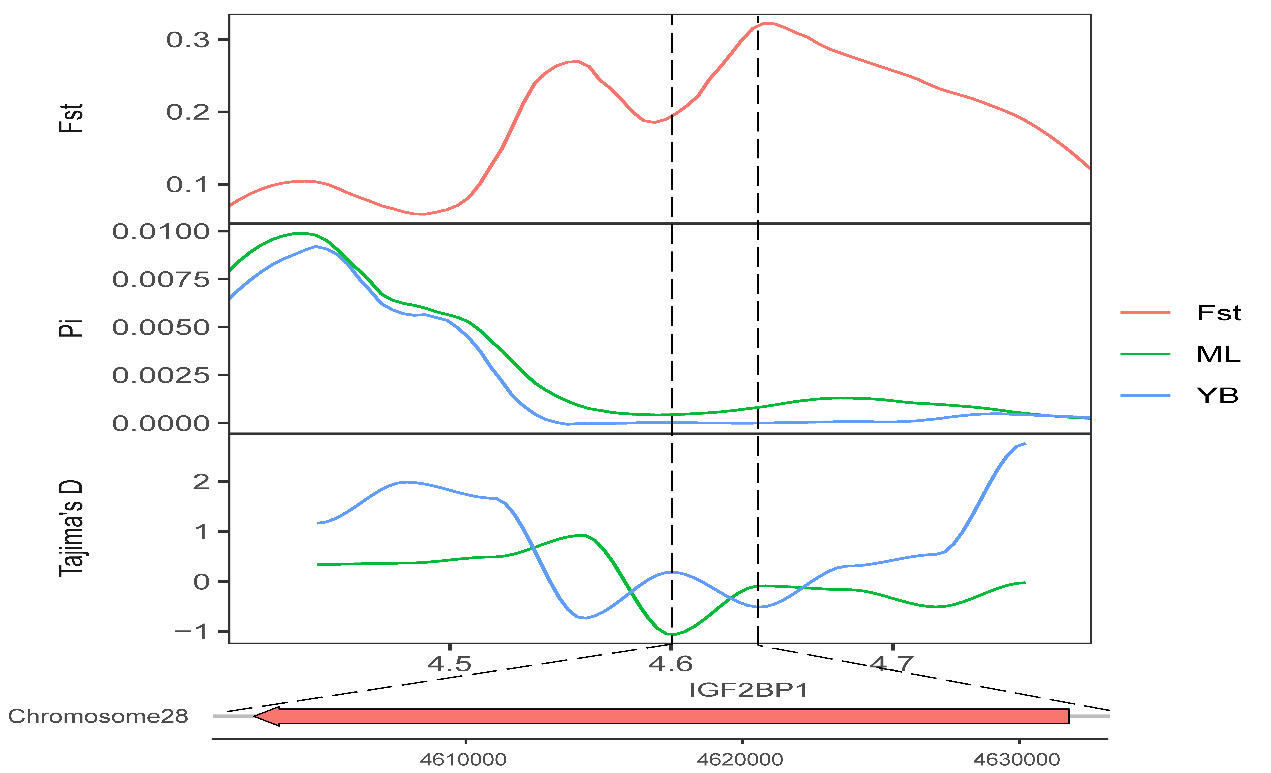


**Figure S6.** Fst、Pi and Tajima’s D value of *IGF2BP1* between Jianshui Yellow-brown duck and Mallard.


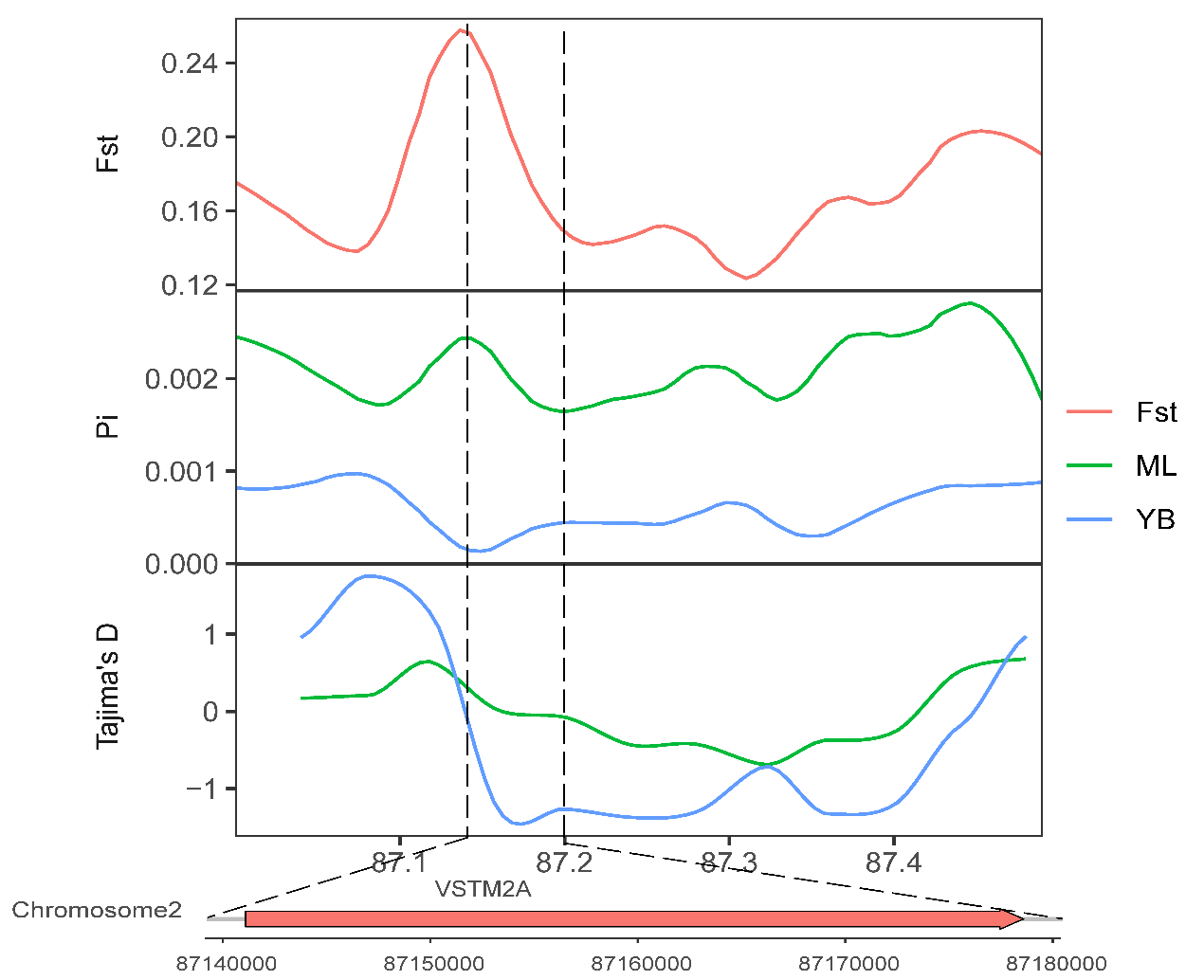


**Figure S7.** Fst、Pi and Tajima’s D value of *VSTM2A* between Jianshui Yellow-brown duck and Mallard.


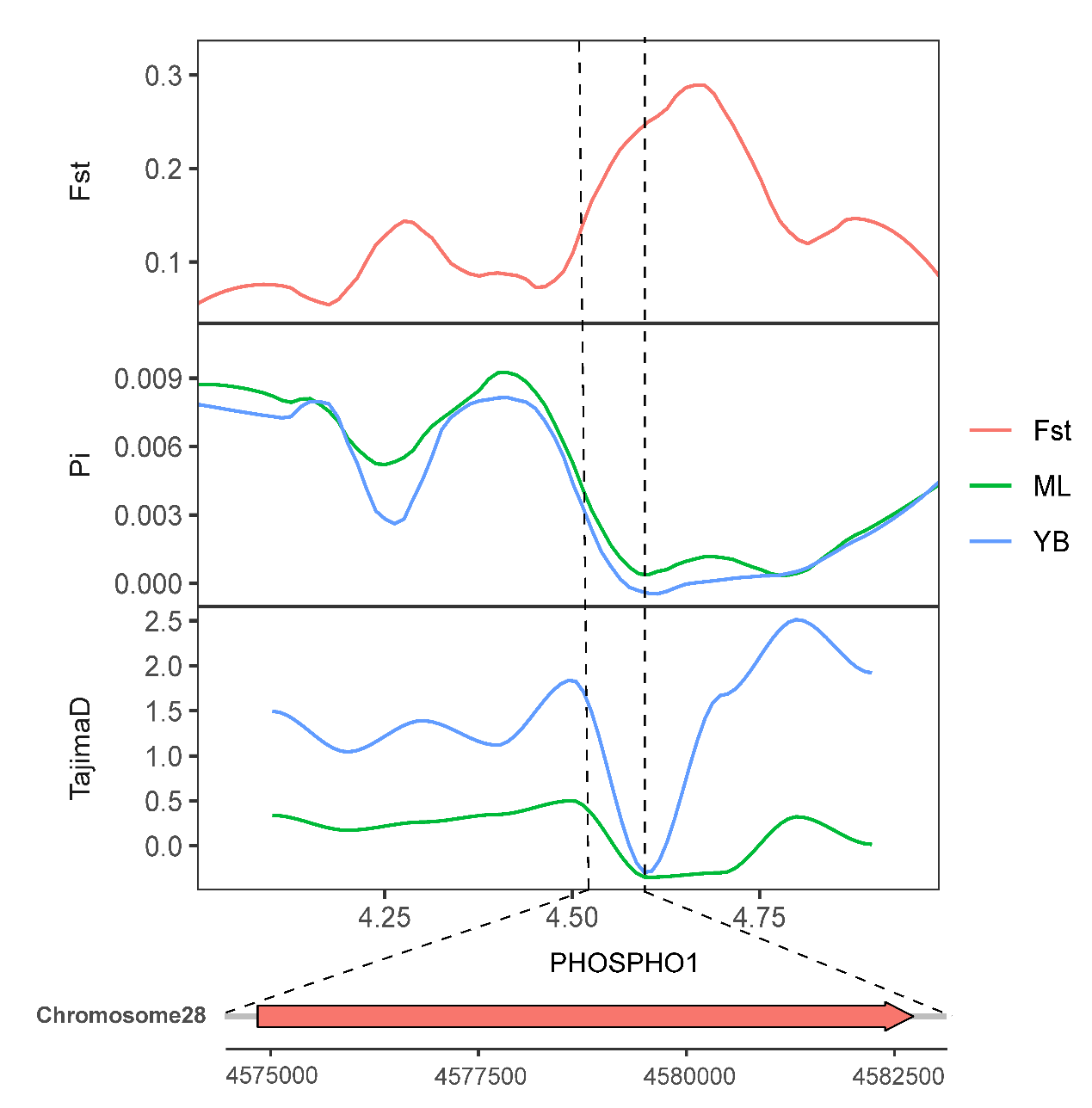


**Figure S8.** Fst、Pi and Tajima’s D value of *PHOSPHO1* between Jianshui Yellow-brown duck and Mallard.


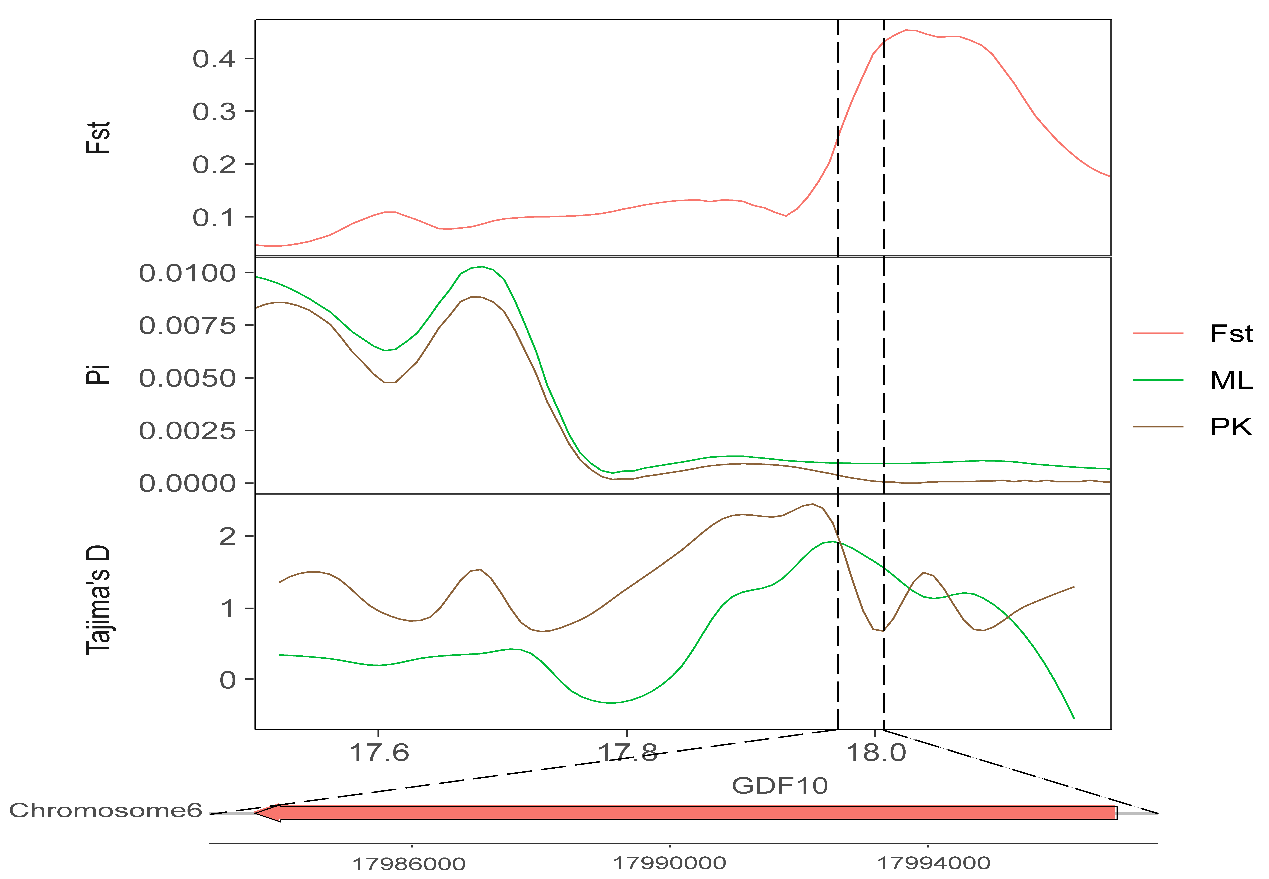


**Figure S9.** Fst、Pi and Tajima’s D value of *GDF10* between Pekin duck and Mallard.


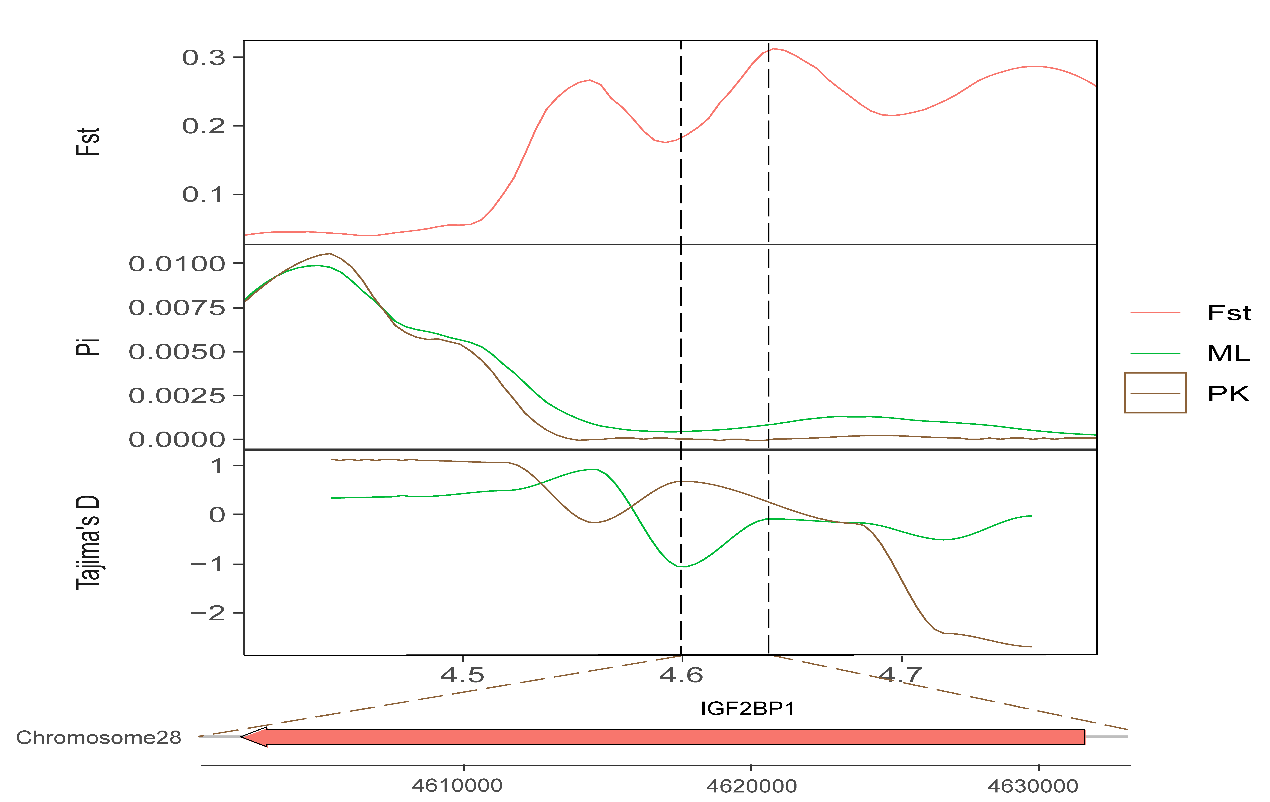


**Figure S10.** Fst、Pi and Tajima’s D value of *IGF2BP1* between Pekin duck and Mallard.


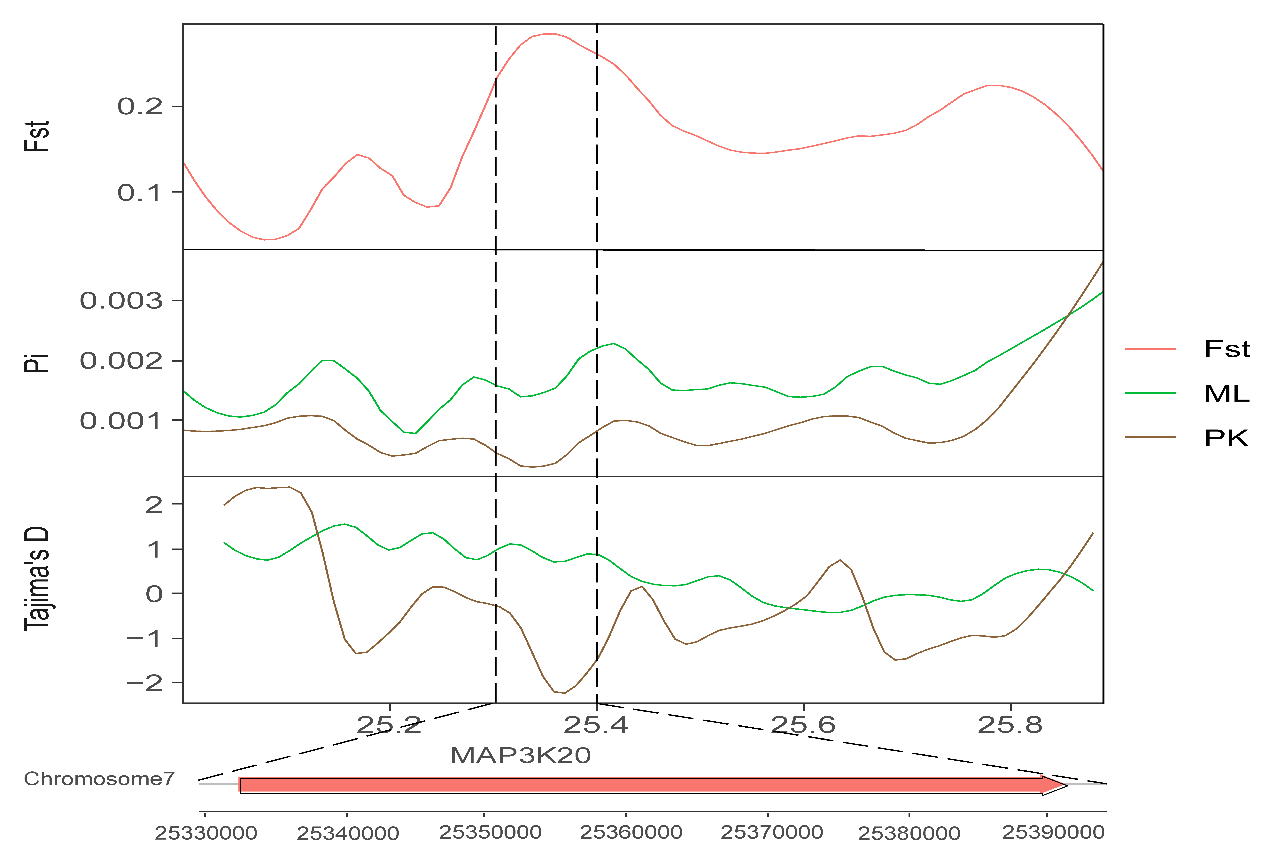


**Figure S11.** Fst、Pi and Tajima’s D value of *MAP3K20* between Pekin duck and Mallard.


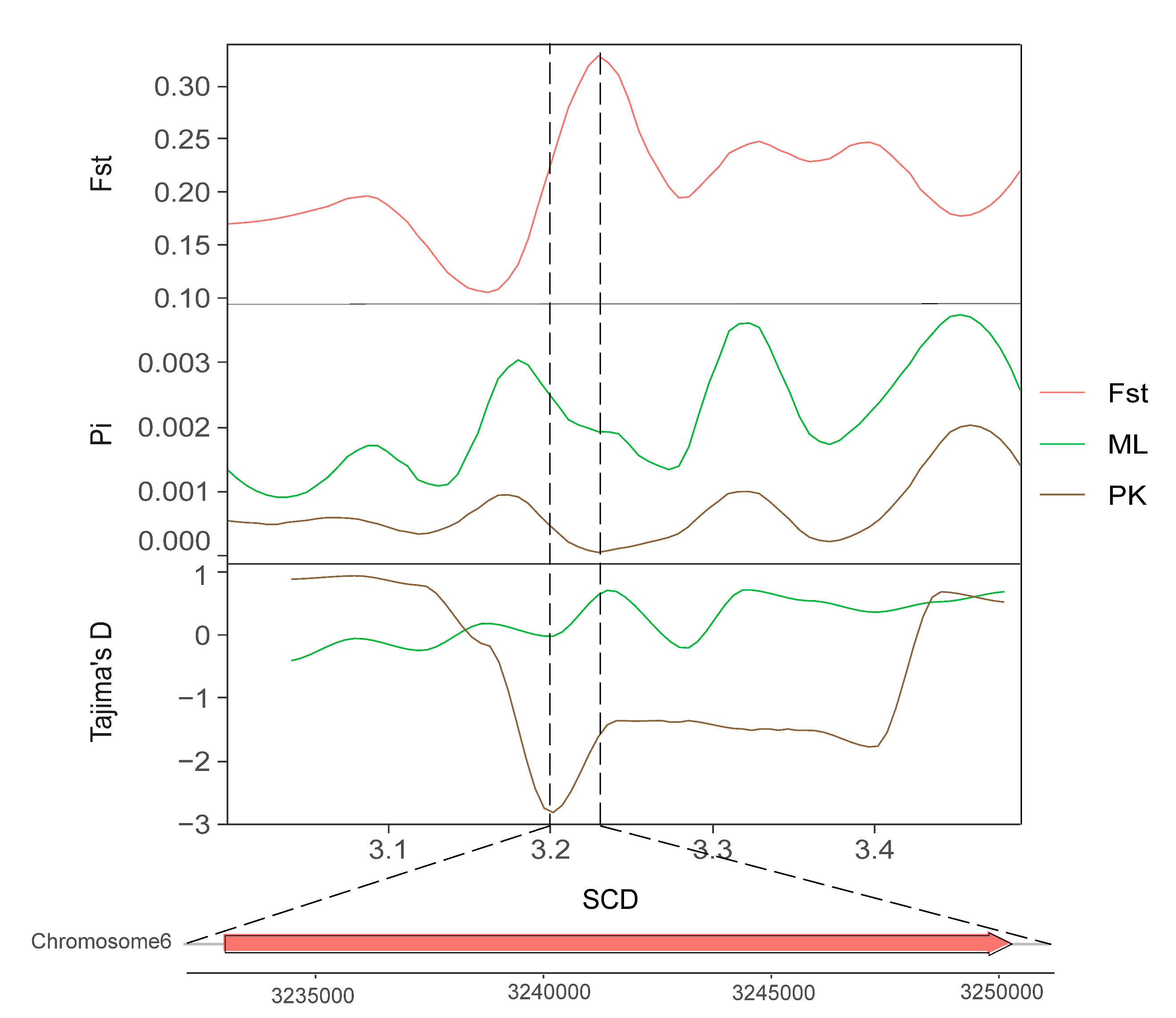


**Figure S12.** Fst、Pi and Tajima’s D value of SCD between Pekin duck and Mallard.


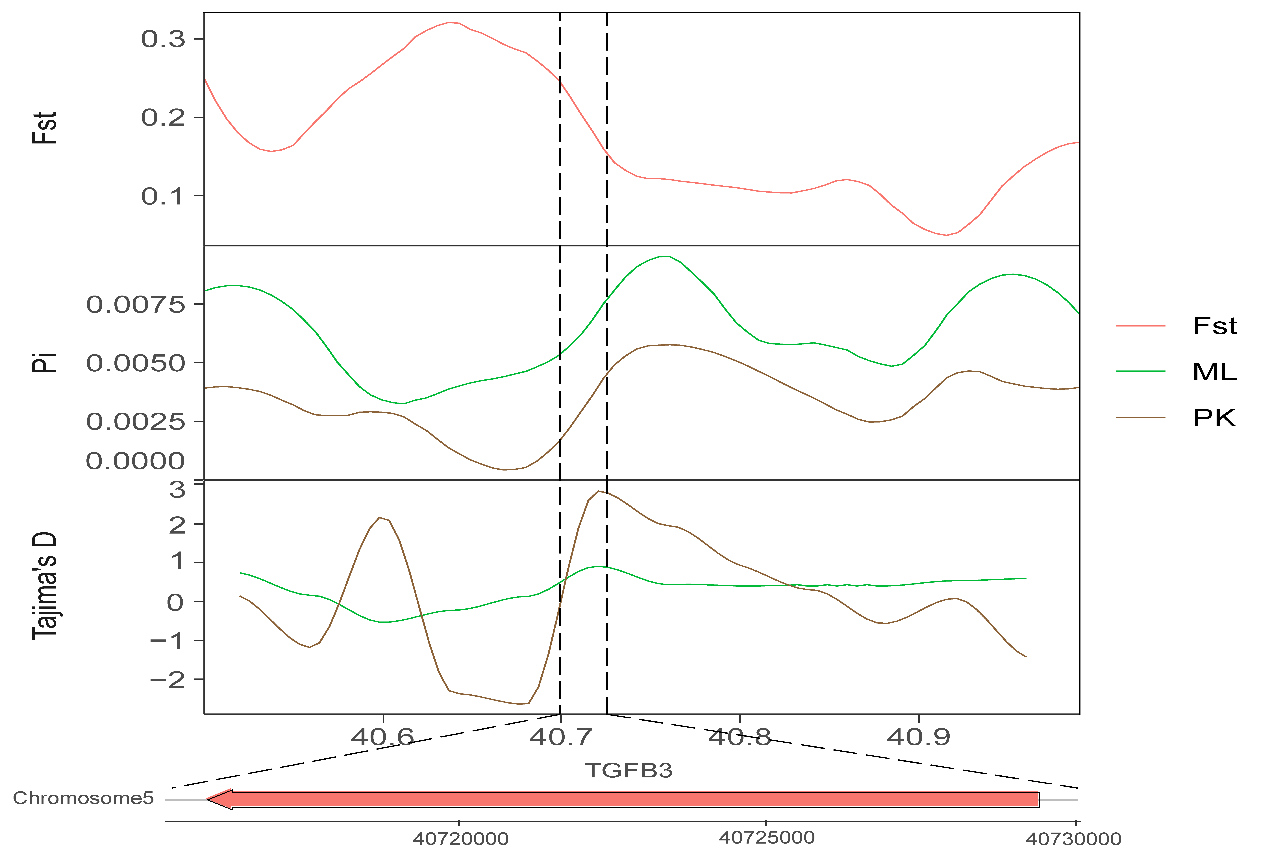


**Figure S13.** Fst、Pi and Tajima’s D value of *TGFB3* between Pekin duck and Mallard.


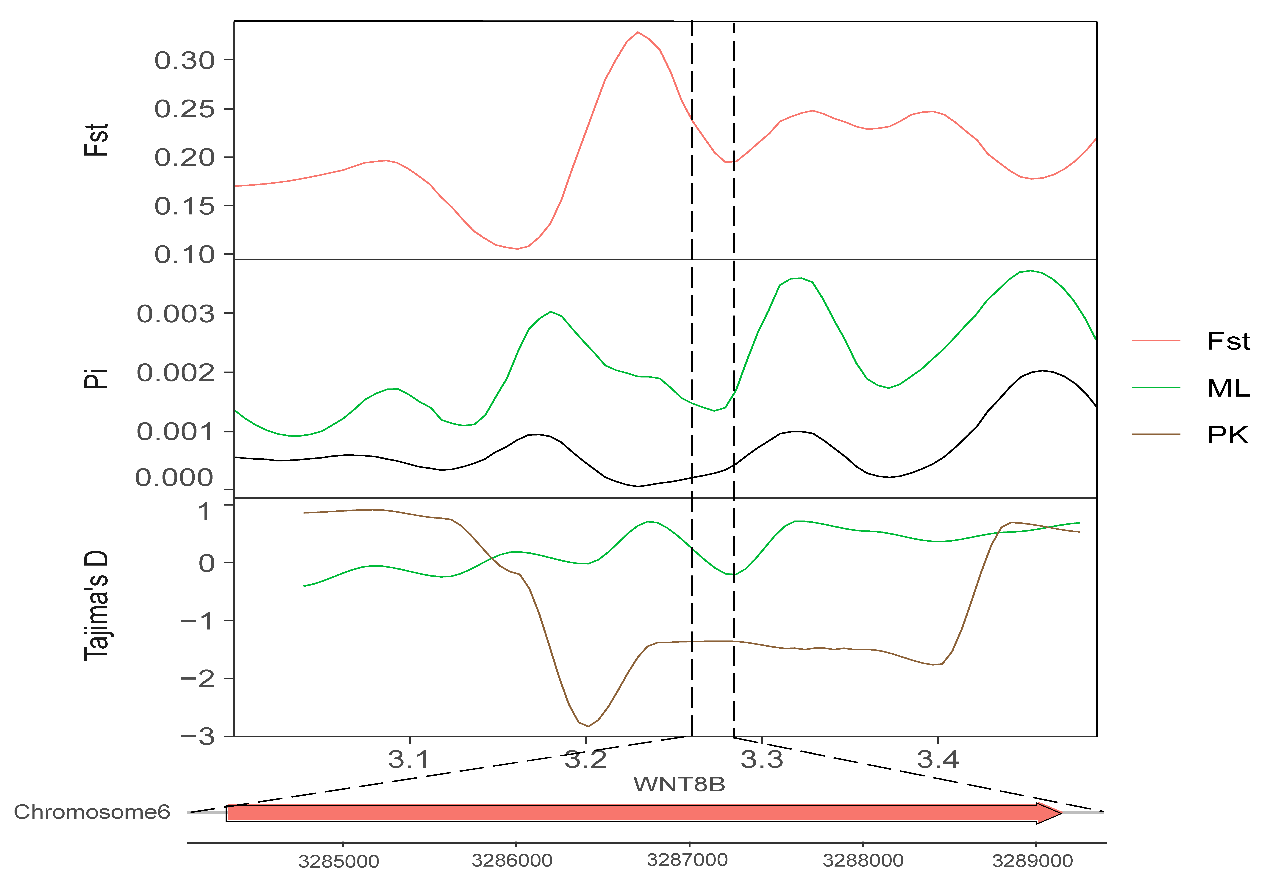


**Figure S14.** Fst、Pi and Tajima’s D value of *WNT8B* between Pekin duck and Mallard.


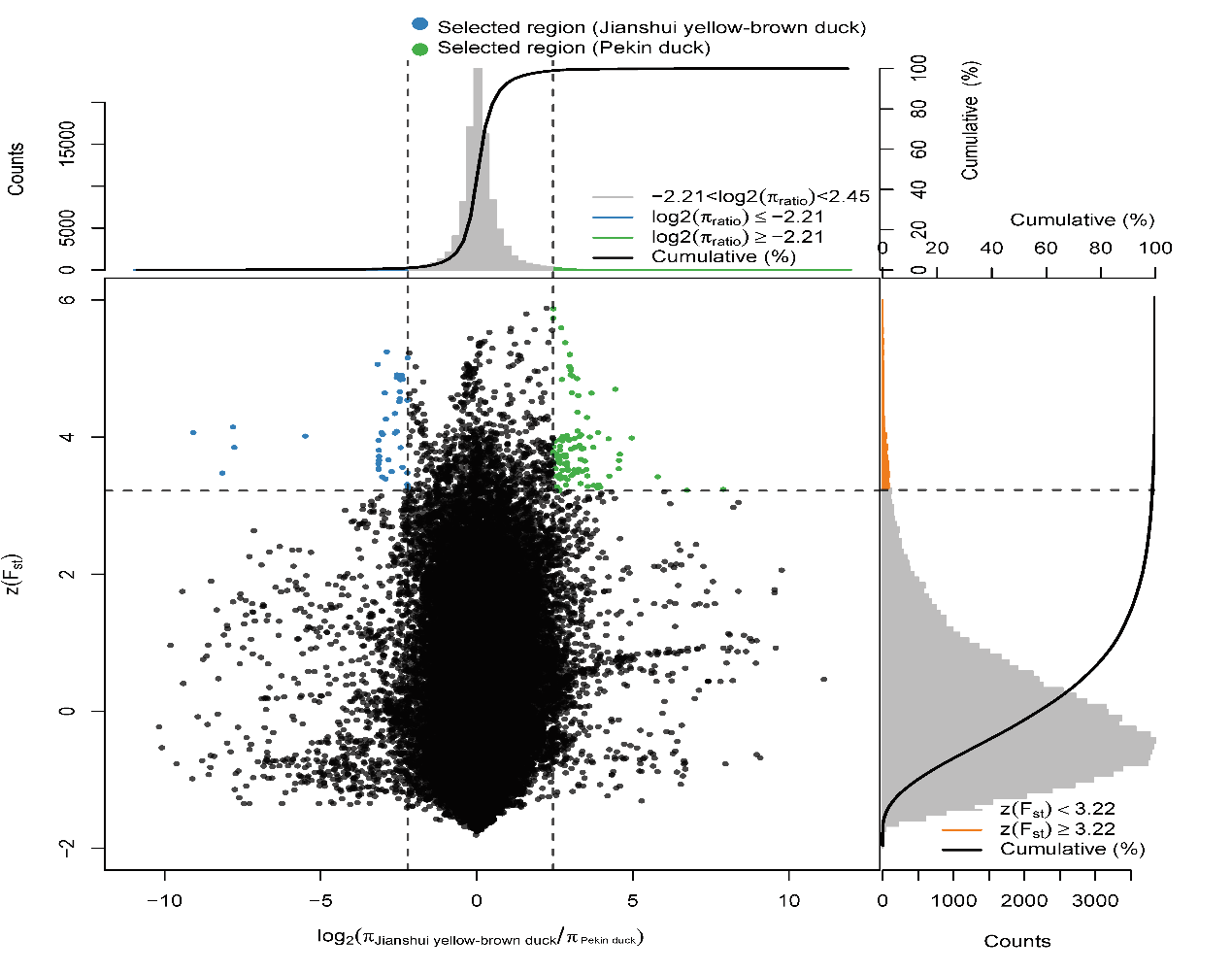


**Figure S15.** Distribution of Z(FST)values and log2(π_Jianshui Yellow-brown duck_/π_Pekin duck_) calculated in 40-kb sliding windows with 10-kb overlap between Jianshui yellow-brown duck and Mallard. Green dots represent selected regions on the genome of Jianshui yellow-brown duck, blue dots represent selected regions on the genome of Mallard.


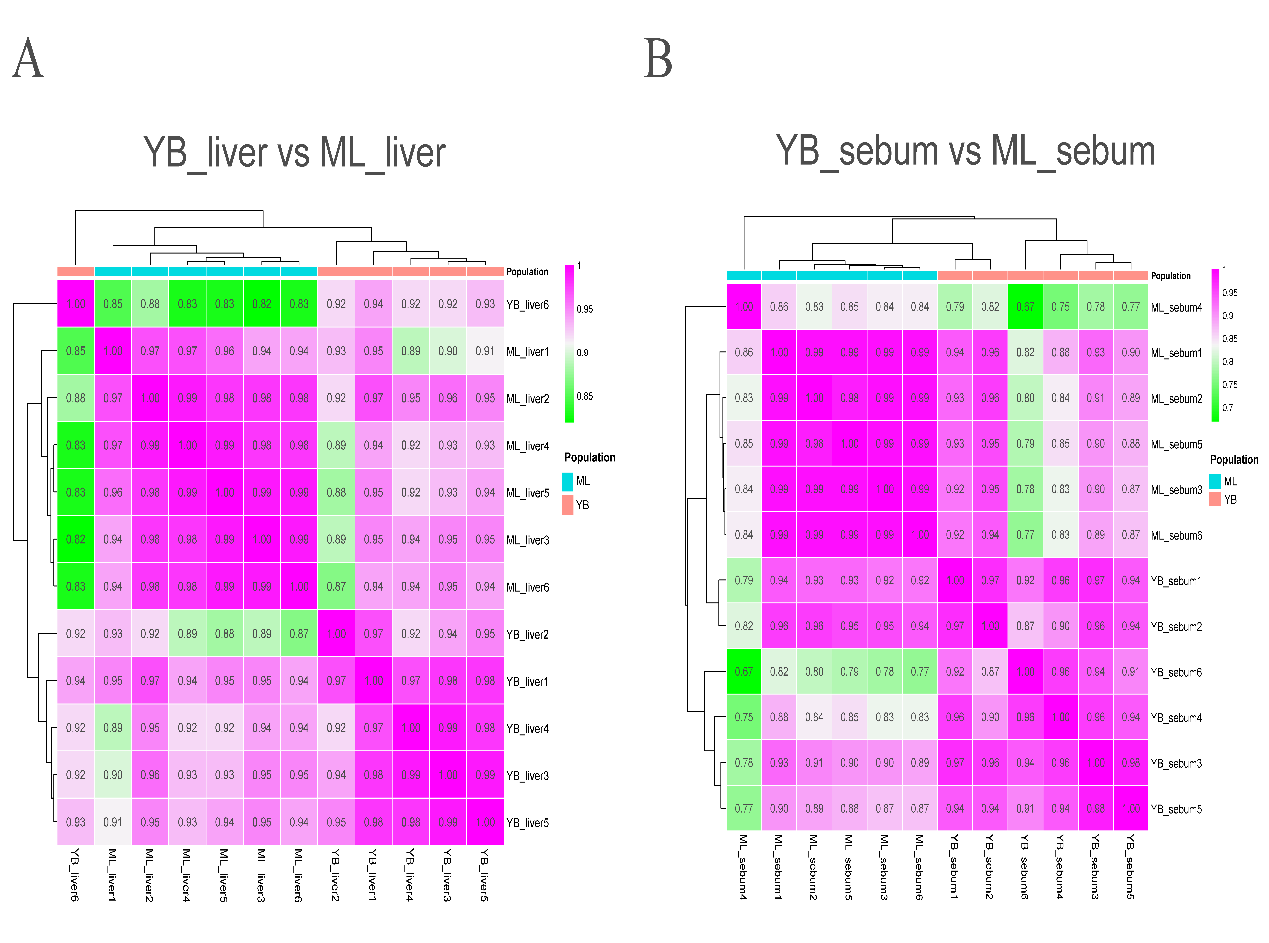


**Figure S16.** Correlation between liver and sebum tissue samples of Jianshui yellow-brown duck and Mallards. (**A**) Correlation of transcriptome sequencing samples from liver tissues of Jianshui yellow-brown ducks and Mallards. (**B**) Correlation of transcriptome sequencing samples from sebum tissue of Jianshui yellow-brown ducks and Mallards.


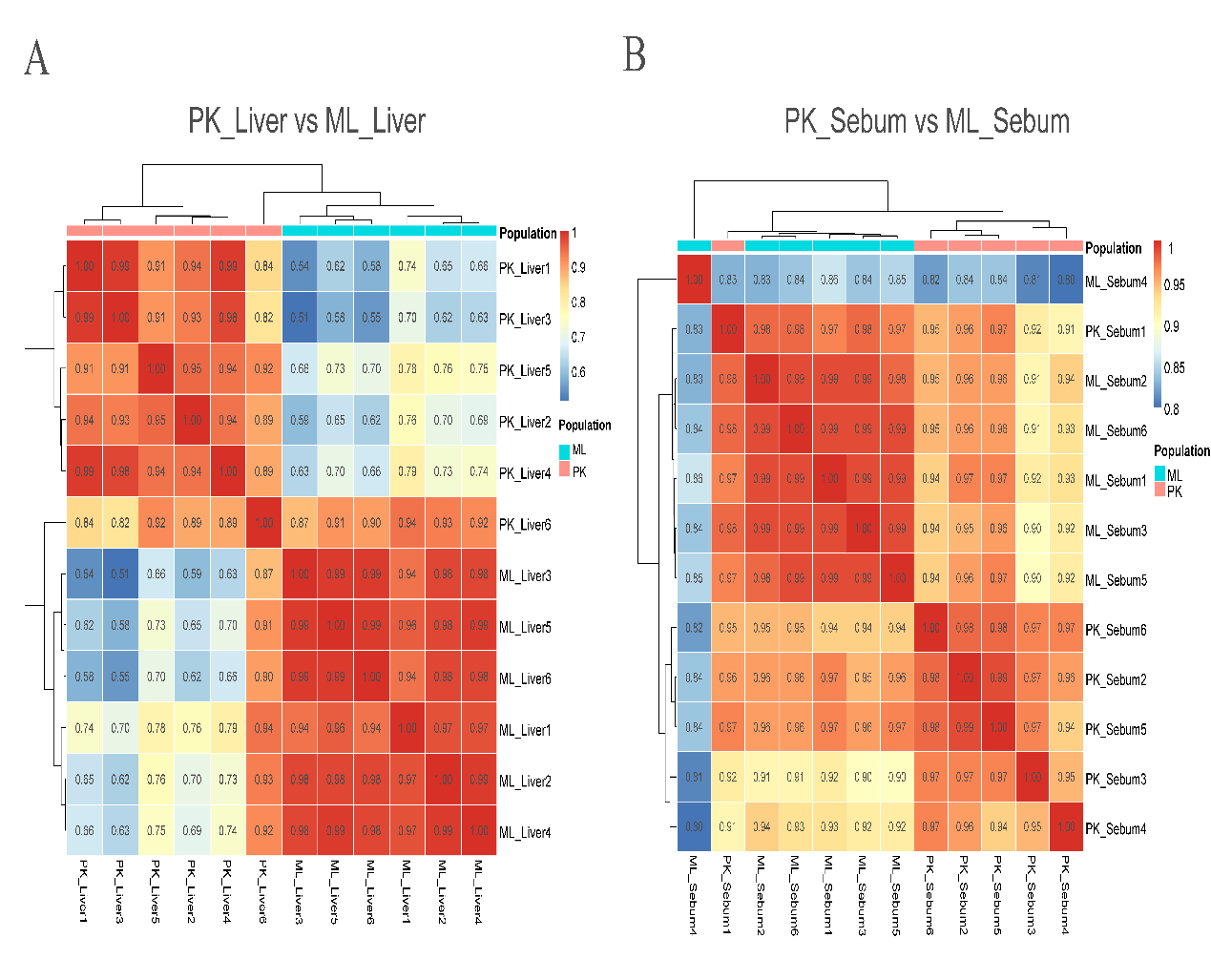


**Figure S17.** Correlation between liver and sebum tissue samples of Pekin ducks and Mallards. (**A**) Correlation of transcriptome sequencing samples from liver tissues of Pekin ducks and Mallards. (**B**) Correlation of transcriptome sequencing samples from sebum tissue of Pekin ducks and Mallards.


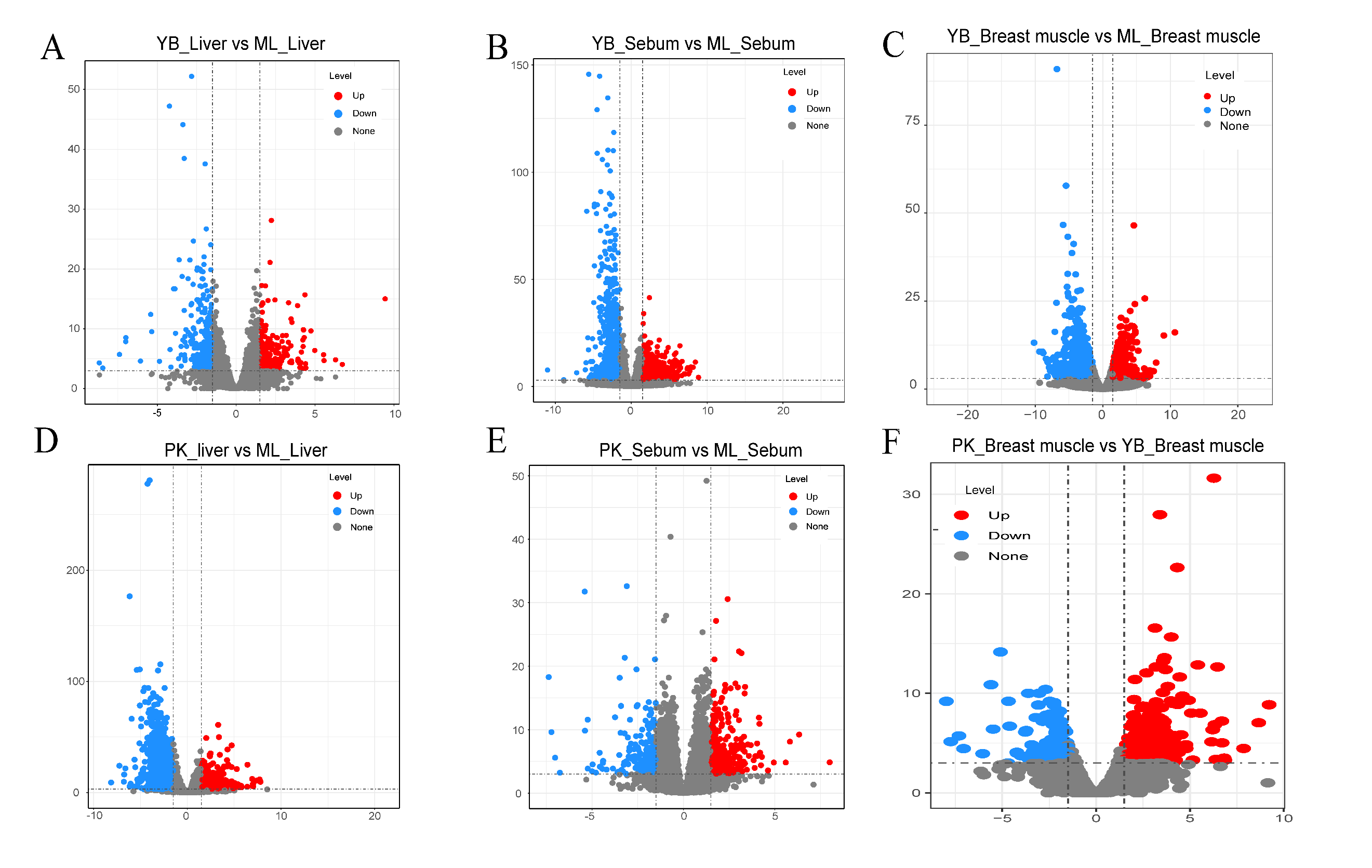


**Figure S18.** Volcanoes of differentially expressed genes, red dots indicate up-regulation of genes, blue dots indicate down-regulation of genes, and gray dots indicate insignificant differences. (A) Differentially expressed genes in liver of Jianshui yellow-brown duck and Mallard. (B) Differentially expressed genes in sebum of Jianshui yellow-brown and Mallard. (C) Differentially expressed genes in liver of Pekin and Mallard. (D) Differentially expressed genes in sebum of Pekin and Mallard.
